# Inhalation delivery of dexamethasone with iSEND nanoparticles attenuates the COVID-19 cytokine storm in mice and nonhuman primates

**DOI:** 10.1126/sciadv.adg3277

**Published:** 2023-06-14

**Authors:** Qian-Fang Meng, Wanbo Tai, Mingyao Tian, Xinyu Zhuang, Yuanwei Pan, Jialin Lai, Yangtao Xu, Zhiqiang Xu, Min Li, Guangyu Zhao, Guang-Tao Yu, Guocan Yu, Rongchang Chen, Ningyi Jin, Xiao Li, Gong Cheng, Xiaoyuan Chen, Lang Rao

**Affiliations:** ^1^ Institute of Biomedical Health Technology and Engineering, Shenzhen Bay Laboratory, Shenzhen 518132, China.; ^2^ Institute of Infectious Diseases, Shenzhen Bay Laboratory, Shenzhen 518132, China.; ^3^ Tsinghua-Peking Center for Life Sciences, School of Medicine, Tsinghua University, Beijing 100084, China.; ^4^ Changchun Veterinary Research Institute, Chinese Academy of Agricultural Sciences, Changchun 130122, China.; ^5^ Departments of Diagnostic Radiology, Surgery, Chemical and Biomolecular Engineering, and Biomedical Engineering, Yong Loo Lin School of Medicine and College of Design and Engineering, National University of Singapore, Singapore 119074, Singapore.; ^6^ State Key Laboratory of Pathogen and Biosecurity, Beijing Institute of Microbiology and Epidemiology, Beijing 100071, China.; ^7^ Stomatological Hospital, Southern Medical University, Guangzhou 510280, China.; ^8^ Department of Chemistry, Tsinghua University, Beijing 100084, China.; ^9^ Institute of Respiratory Disease, Shenzhen People’s Hospital, Shenzhen 518020, China.; ^10^ Nanomedicine Translational Research Program, NUS Center for Nanomedicine, Yong Loo Lin School of Medicine, National University of Singapore, Singapore 117597, Singapore.; ^11^ Clinical Imaging Research Centre, Centre for Translational Medicine, Yong Loo Lin School of Medicine, National University of Singapore, Singapore 117599, Singapore.; ^12^ Institute of Molecular and Cell Biology, Agency for Science, Technology, and Research (A*STAR), Singapore 138673, Singapore.

## Abstract

Dexamethasone (DEX) is the first drug to show life-saving efficacy in patients with severe coronavirus disease 2019 (COVID-19), while DEX is associated with serious adverse effects. Here, we report an inhaled, Self-immunoregulatory, Extracellular Nanovesicle-based Delivery (iSEND) system by engineering neutrophil nanovesicles with cholesterols to deliver DEX for enhanced treatment of COVID-19. Relying on surface chemokine and cytokine receptors, the iSEND showed improved targeting to macrophages and neutralized broad-spectrum cytokines. The nanoDEX, made by encapsulating DEX with the iSEND, efficiently promoted the anti-inflammation effect of DEX in an acute pneumonia mouse model and suppressed DEX-induced bone density reduction in an osteoporosis rat model. Relative to an intravenous administration of DEX at 0.1 milligram per kilogram, a 10-fold lower dose of nanoDEX administered by inhalation produced even better effects against lung inflammation and injury in severe acute respiratory syndrome coronavirus 2–challenged nonhuman primates. Our work presents a safe and robust inhalation delivery platform for COVID-19 and other respiratory diseases.

## Teaser

An inhaled, Self-immunoregulatory, Extracellular Nanovesicle-based Delivery (iSEND) system has been developed to treat COVID-19.

## INTRODUCTION

The coronavirus disease 2019 (COVID-19) pandemic, induced by the severe acute respiratory syndrome coronavirus 2 (SARS-CoV-2) (*1*, *2*), has resulted in broad consequences for public health and global economy (*3*, *4*). Mechanically, in response to the SARS-CoV-2 infection, the body stimulates immunocyte activation and cytokine secretion to eliminate viruses and inhibit infection (*5*). However, sustainably high levels of cytokines, characterized as “cytokine storm,” may, in turn, aggravate the inflammation state and lead to immune dysfunction (*6*, *7*). Clinically, most patients with COVID-19 show mild or moderate symptoms, but ~20% of patients progress to severe pneumonia, multiple organ failure, and even septic shock owing to the cytokine storm (*8*). Despite the rapid progress made toward effective control of COVID-19 (*9*–*11*), particularly the very recent encouraging outcomes of oral drugs molnupiravir and Paxlovid in treating mild or moderate cases (*12*), there are only few available drugs or therapeutic strategies for patients with severe COVID-19 (*13*).

Dexamethasone (DEX) is the first drug to show life-saving efficacy in patients with severe COVID-19 (*14*). In the world’s largest clinical trial involving COVID-19, the use of DEX reduces the number of COVID-19–mediated deaths by 35 and 20% in patients who require mechanical ventilation and in nonventilated patients with oxygen treatment, respectively (*15*). Moreover, the DEX treatment resulted in a higher hospital discharge rate and in a shorter hospitalization time (*15*). DEX is expected to have significant impact on COVID-19, not only because DEX is the first drug to significantly improve patient survival (*16*) but also because it is widely available and very cheap (*14*, *16*). Although promising, DEX is a glucocorticoid and its receptors are widely expressed in most cell types; thus, the use of DEX may lead to severe side effects (*16*, *17*), such as osteoporosis and fracture, femoral head ischemic necrosis, and central obesity, which limit its widespread application, particularly for long-term treatments at high doses (*18*, *19*). Despite these concerns, the broad action of DEX on suppressing cytokines may be beneficial in this special situation (*15*, *16*), especially if DEX can be delivered to lung tissues and target cells that play a key role in the acute and progressive phase of COVID-19 (*16*).

Extracellular vesicles (EVs) are lipid vesicles secreted by cells and involved in physiological and pathological processes (*20*–*22*) and, thus, have great potential in immunotherapy (*22*, *23*). Moreover, benefiting from their desirable safety and stability, the EVs have been regarded as a next-generation drug delivery platform (*24*–*27*). In terms of production, however, the EVs secreted by cells are insufficient for use in immunotherapy and drug delivery (*24*, *28*). As a result, cell-derived nanovesicles (NVs), which can be prepared by sonication and extrusion of cellular membranes, have been used as an alternative to EVs (*29*–*31*). The NVs also contain lipids and specific proteins of source cells and thus inherit unique properties from source cells (*32*). For example, we and others have recently demonstrated that NVs could serve as decoys to trap viruses and broad-spectrum cytokines (*33*, *34*), inhibiting viral infection and lung inflammation (*35*, *36*). In this work, we propose to use NVs to improve COVID-19 treatment by targeted delivery of DEX and synergetic neutralization of cytokines.

**Fig. 1.**
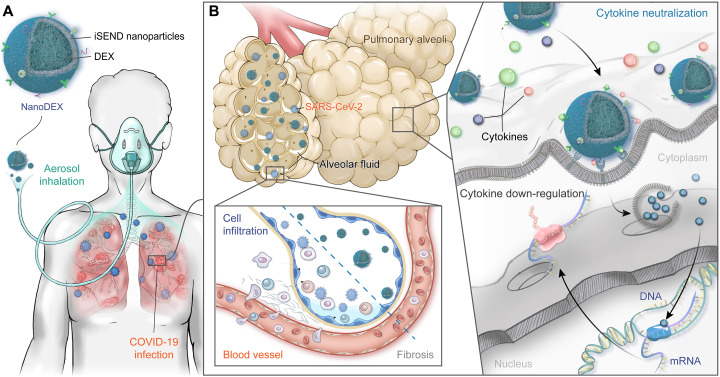
Schematic illustration of inhalable nanoDEX for COVID-19. (**A**) The inhalable nanoDEX is composed of iSEND nanoparticles loaded with DEX. Benefiting from inhalational delivery and chemokine receptors on the iSEND, the nanoDEX shows enhanced retention in inflamed lungs and promotes targeting to alveoli immunocytes. (**B**) The nanoDEX protects against COVID-19 cytokine storm through a powerful two-step strategy: cytokine down-regulation by DEX in the first step followed by cytokine neutralization by the iSEND in the second step. Enhanced retention of nanoDEX in inflamed lungs promotes cytokine down-regulation effects of DEX, thus suppressing the cell infiltration and lung fibrosis caused by the COVID-19 infection. Moreover, depending on specific cytokine receptors on the iSEND, the nanoDEX binds and neutralizes broad-spectrum cytokines, further improving the COVID-19 treatment and complication management.

A recent report has revealed that the use of DEX in patients with severe COVID-19 affected circulating neutrophils, altered interferon (IFN)^active^ neutrophils, and activated interleukin-1 receptor 2 (IL-1R2)^+^ neutrophils (*13*). Inspired by this, an inhalable DEX nanoformulation (nanoDEX) comprising neutrophil-derived NVs (N-NVs) loaded with DEX (DEX-N-NVs) was developed to improve the COVID-19 treatment and complication management (Fig. 1A). By taking advantages of inhalation delivery and abundant chemokine receptors on N-NVs (*37*), the nanoDEX shows enhanced retention in inflamed lungs and improved targeting to activated macrophages and dendritic cells (DCs). The nanoDEX protects against the COVID-19 cytokine storm through a powerful two-step strategy: cytokine down-regulation in the first step followed by cytokine neutralization in the second step (Fig. 1B). Enhanced retention of nanoDEX in lungs promotes the cytokine down-regulation effects of DEX, thus suppressing inflammatory cell infiltration and lung injury caused by the SARS-CoV-2 infection. Moreover, relying on abundant cytokine receptors on N-NVs, the nanoDEX binds and neutralizes broad-spectrum cytokines, improving the COVID-19 treatment efficacy. In addition, because of the enhanced retention in inflamed lungs, the nanoDEX treatment significantly attenuates DEX-induced osteoporosis, further highlighting the significance of nanoDEX.

## RESULTS

### DEX-N-NVs contained abundant cytokine receptors and showed sustained drug release

**Fig. 2.**
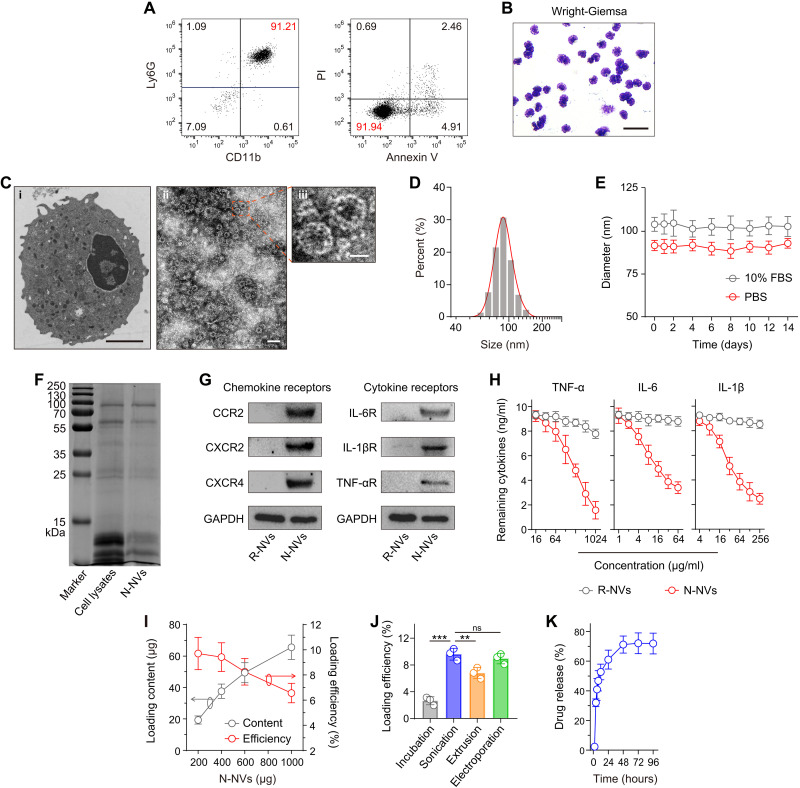
Preparation and characterization of DEX-N-NVs. (**A**) Flow cytometric analysis of neutrophil purity and apoptosis. (**B**) Wright-Giemsa staining of neutrophils. Scale bar, 50 μm. (**C**) Transmission electron microscopy (TEM) images of (i) neutrophils and (ii and iii) N-NVs. Scale bars, (i) 2 μm, (ii) 200 nm, and (iii) 50 nm. (**D**) Size distribution of N-NVs. (**E**) Stability of N-NVs in phosphate-buffered saline (PBS) and 10% fetal bovine serum (FBS) over 14 days. (**F**) SDS-PAGE protein analysis of neutrophil lysates and N-NVs. (**G**) Western blot analysis of chemokine and cytokine receptors including C-C motif chemokine receptor 2 (CCR2), C-X-C motif chemokine receptor 2 (CXCR2), CXCR4, IL-6 receptor (IL-6R), IL-1βR, and tumor necrosis factor–α receptor (TNF-αR) in red blood cell–derived NVs (R-NVs) and N-NVs. (**H**) Binding capacity analysis of R-NVs and N-NVs with TNF-α, IL-6, and IL-1β. (**I**) Drug loading content and efficiency of DEX into N-NVs after sonication. (**J**) Drug loading efficiency with different loading strategies. The initial N-NVs are 200 μg. (**K**) Drug release profile of DEX-N-NVs. All data are presented as means ± SD (*n* = 3). Statistical significance was calculated via ordinary one-way analysis of variance (ANOVA) with Dunnett’s test (J). **P < 0.01 and ***P < 0.001. GAPDH, glyceraldehyde-3-phosphate dehydrogenase; ns, not significant.

The preparation of DEX-N-NVs includes three steps: (i) isolating neutrophils, (ii) obtaining N-NVs, and (iii) loading DEX into N-NVs. First, we extracted mature neutrophils from mouse/rhesus bone marrow and purified them by density gradient centrifugation (*38*). Flow cytometric analysis revealed a 91.21% purity of isolated neutrophils (Ly6G^+^/CD11b^+^) (Fig. 2A and fig. S1), with only a small fraction of cells undergoing early apoptosis [annexin V^+^/propidium iodide (PI)^−^] and late apoptosis (annexin V^+^/PI^+^) (Fig. 2A). Furthermore, Wright-Giemsa staining showed a purple color typical of neutral substances and polymorphonuclear morphology in neutrophils (Fig. 2B).

To obtain N-NVs, the intracellular content of isolated neutrophils was removed by hypotonic lysis, mechanical disruption, and gradient centrifugation. N-NVs were prepared by serial sonication and extrusion of cell membranes through nanopores with a mini extruder. Transmission electron microscopy (TEM) visualization and dynamic light scattering (DLS) analysis revealed that N-NVs were round lipid droplets with an average size of 90 nm (Fig. 2, C and D, and fig. S2). Notably, N-NVs remained stable over at least 14 days in physiological buffers (Fig. 2E and fig. S3) and showed no significant cytotoxicity (fig. S4), ensuring downstream in vitro and in vivo experiments.

SDS–polyacrylamide gel electrophoresis (SDS-PAGE) suggested that N-NVs inherited specific protein contents from neutrophils (Fig. 2F). Furthermore, Western blotting confirmed the existence of key chemokine and cytokine receptors on N-NVs, but not on red blood cell–derived NVs (R-NVs) (Fig. 2G), including C-C motif chemokine receptor 2 (CCR2), C-X-C motif chemokine receptor 2 (CXCR2), CXCR4, IL-6 receptor (IL-6R), IL-1βR, and tumor necrosis factor–α receptor (TNF-αR). Relying on these cytokine receptors, N-NVs efficiently adsorbed inflammatory cytokines (Fig. 2H), including IL-6, IL-1β, and TNF-α in a dose-dependent manner, indicating the potential of N-NVs on cytokine storm suppression. Notably, compared with other hemocyte- and immunocyte-derived NVs, N-NVs showed better performance on IL-6 neutralization (fig. S5).

After that, we prepared DEX-N-NVs by sonication, loading DEX into the resulting N-NVs. The drug loading efficiency was determined by high-performance liquid chromatography (HPLC) (fig. S6), and DEX-N-NVs with an optimal loading efficiency of approximately 9.71% was prepared under an initial feeding ratio of 1:5 (Fig. 2I). We also tested the influence of loading strategies on the efficiency and found that sonication and electroporation were better for DEX-N-NV preparation compared with incubation and extrusion (Fig. 2J). Notably, the sonication loading of DEX did not alter the size of N-NVs, cytokine receptors, and cytokine neutralization (fig. S7). Moreover, approximately 71.16% of total DEX leaked out from N-NVs within 48 hours (Fig. 2K), suggesting a sustained release profile of DEX-N-NVs.

### DEX-N-NVs showed enhanced targeting to inflamed cells and improved anti-inflammation effect in vitro

**Fig. 3.**
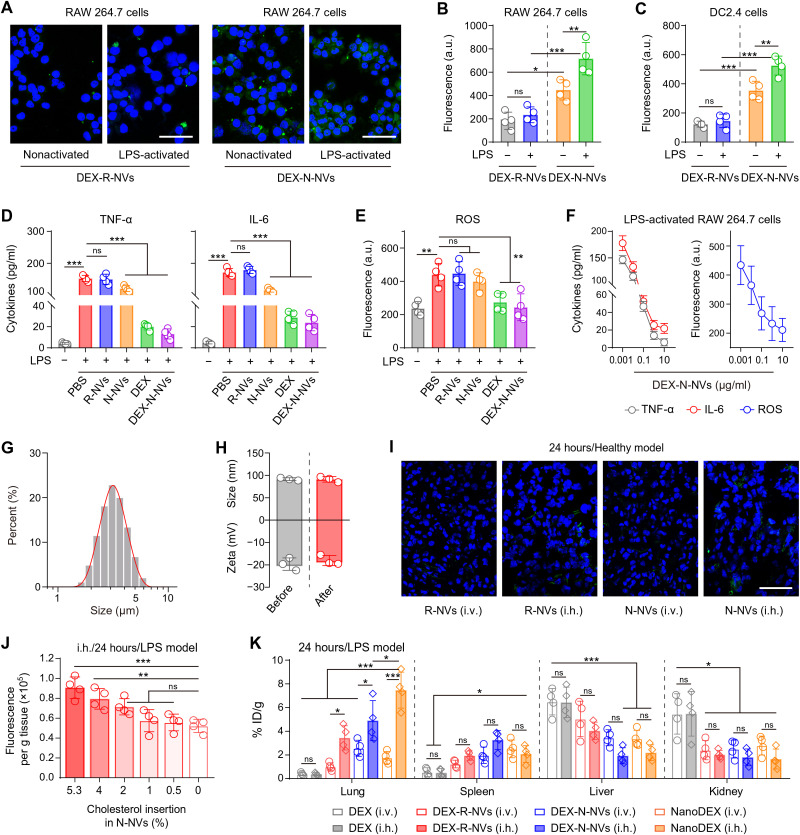
The nanoDEX showed enhanced targeting to inflamed cells and improved retention in inflamed lungs. (**A**) Fluorescence images of nonactivated or LPS-activated RAW 264.7 cells after incubation with DEX-R-NVs or DEX-N-NVs. Scale bars, 100 μm. DEX-R-NVs and DEX-N-NVs are labeled with 3,3′-dioctadecyloxacarbocyanine perchlorate (DiO) (green) before the incubation. (**B** and **C**) Fluorescence intensity analysis of DiO (green) in (B) RAW 264.7 and (C) DC2.4 cells after indicated treatments. (**D**) TNF-α, IL-6, and (**E**) ROS levels in the supernatant of LPS-stimulated RAW 264.7 cells after indicated treatments. (**F**) Dose-dependent effects of DEX-N-NVs on TNF-α, IL-6, and ROS expression in the supernatant of LPS-stimulated RAW 264.7 cells. (**G**) Size distribution of the droplets containing DEX-N-NVs after inhalation delivery. (**H**) Hydrodynamic diameter and zeta potential of DEX-N-NVs before and after inhalation delivery. (**I**) Fluorescent images showing the lung accumulation of R-NVs or N-NVs after intravenous or inhalation delivery. Scale bars, 50 μm. R-NVs and N-NVs are labeled with DiO (green) before inhalation delivery. (**J**) Lung accumulation of cholesterol-engineered N-NVs at 24 hours after inhalation delivery in LPS-infected mice. (**K**) Biodistribution of DEX, DEX-R-NVs, DEX-N-NVs, or nanoDEX in major organs at 24 hours after intravenous or inhalation delivery in LPS-infected mice. All data are presented as means ± SD (*n* = 4). Statistical significance was calculated via ordinary one-way ANOVA with Tukey’s test (B, C, and K) or Dunnett’s test (D, E, and J). *P < 0.05, **P < 0.01, and ***P < 0.001. a.u., arbitrary units; i.h., inhalation; i.v., intravenous.

Macrophages and DCs are known to engage in SARS-CoV-2 infection and the COVID-19 cytokine storm, resulting in their activation and excessive secretion of inflammatory cytokines (*5*). Here, DEX-N-NVs were fluorescently labeled and incubated with RAW 264.7 macrophage-like and DC2.4 DC-like cells. R-NVs loaded with DEX (DEX-R-NVs) were tested as a control because they had similar structures as DEX-N-NVs, but red blood cells had less cytokine receptors compared with neutrophils. Significant fluorescence was observed on the cells after incubation with DEX-N-NVs, but not with DEX-R-NVs (Fig. 3, A to C, and fig. S8). Lipopolysaccharide (LPS)–activated cells, when incubated with DEX-N-NVs, showed a significant fluorescence increase compared with naive cells (Fig. 3, B and C). Furthermore, the treatment by using cytokine blocking significantly decreased the targeting of DEX-N-NVs to inflamed cells (fig. S9). These results demonstrated the ability of DEX-N-NVs to target inflamed cells, which is attributed to specific interactions between cytokine receptors on N-NVs and cytokine molecules overexpressed by activated macrophages and DCs.

After DEX-N-NVs targeted the inflamed cells, the cells appeared to take up DEX-N-NVs by clathrin-independent pathways including phagocytosis and lipid raft-mediated internalization (*31*), and then DEX was released in the cytoplasm to inhibit the secretion of inflammatory cytokines (*13*). To test the anti-inflammation therapeutic effect of DEX-N-NVs, the LPS was used to stimulate RAW 264.7 cells to mimic viral infection–induced inflammatory states in vitro (*35*). After the stimulation, the secretion of TNF-α and IL-6 was significantly improved (Fig. 3D), confirming the activated inflammatory states. The production of IL-1β was not affected obviously, probably because of negative regulation of nuclear factor κB signaling in IL-1β secretion (*39*). On the basis of LPS-stimulated RAW 264.7 cells, DEX-N-NVs showed a remarkable anti-inflammation effect in a dose-dependent manner (Fig. 3, D to F), which is probably attributed to a powerful two-step anti-inflammation strategy: cytokine down-regulation by DEX synergized with cytokine neutralization by N-NVs. Moreover, the treatment with DEX-N-NVs reduced the production of reactive oxygen species (ROS) (Fig. 3, E and F), suggesting effective remission of systemic inflammation.

### The iSEND (cholesterol-engineered N-NVs) showed enhanced retention in inflamed lungs after inhalation delivery

NVs are lipid vesicles collected from cellular membranes and inherit certain properties from source cells, including immune evasion and regulation (*23*). Especially, relying on their surface CD47 (*40*), N-NVs can effectively escape the immune clearance and realize their accumulation in inflamed lungs. To investigate the in vivo performance of DEX-N-NVs, the mice received inhalation delivery of phosphate-buffered saline (PBS) or PBS containing DEX-N-NVs with a commercially available portable nebulizer. After inhalation delivery, the liquid drops containing DEX-N-NVs were approximately 3 μm in diameter (Fig. 3G), which is suitable for inhalation delivery of specific drugs into pulmonary alveoli (*34*, *36*). Moreover, the inhalation process had no significant effect on the physicochemical property of N-NVs and drug release profile of DEX-N-NVs (Fig. 3H and fig. S10), guaranteeing downstream in vivo experiments with inhalation delivery. After that, the mice received inhalation delivery of fluorescently labeled N-NVs, and the in vivo biodistribution of N-NVs were studied. As expected, inhalation delivery strategy significantly improved the accumulation of N-NVs in the lungs compared with intravenous injection (Fig. 3I). In addition, N-NVs were also detected in the spleen, liver, and kidneys, indicating clearance via the reticuloendothelial system and the metabolization through the body (fig. S11). Given the existence of abundant cytokine receptors on N-NVs and the ability of N-NVs to target inflamed macrophages and DCs in vitro, we further tested the ability of N-NVs to target inflamed lung tissues based on a LPS-induced acute pneumonia model (*35*). As expected, N-NVs showed an enhanced accumulation in inflamed lungs (figs. S12A and `13A). N-NVs could still be found in inflamed lungs 72 hours after inhalation delivery (figs. S12B and S13B), suggesting superior retention of N-NVs.

Considering that NVs are mainly composed of lipids, cholesterols, and proteins and cholesterol plays a key role in the physical stability of cell membranes (*41*), we tried to engineer N-NVs with cholesterol conjugation to further improve the inhalation delivery efficiency. We found that no more than 5.3% of cholesterols could be engineered onto N-NVs, and these cholesterol-engineered N-NVs showed significantly improved accumulation in inflamed lungs (Fig. 3J), which can be attributed to enhanced stability by cholesterols. In the following experiments, we used these N-NVs engineered with 5.3% cholesterols as the iSEND to deliver DEX, and the resulting DEX-iSEND was named as the nanoDEX. We then tested the in vivo performance of the nanoDEX and found that DEX after inhalation delivery showed similar in vivo biodistribution to that after intravenous delivery (Fig. 3K). Notably, benefiting from specific cytokine receptors on N-NVs and improved inhalation delivery stability by cholesterol engineering, the iSEND improved the accumulation of DEX in inflamed lungs for about 14-fold (Fig. 3K). Inhalation delivery of nanoDEX showed superior retention in inflamed lungs (fig. S14), ensuring anti–COVID-19 treatment by our inhalable nanoDEX.

Potential systemic toxicity is always a major concern for drug delivery system (*42*). To investigate the in vivo toxicity of iSEND, the mice received inhalation delivery of PBS or PBS containing iSEND. Neither death nor significant weight difference was observed between the PBS and N-NVs groups over 15 days (fig. S15). Furthermore, serum biochemistry, complete blood test, and histological examination results demonstrated that inhalation delivery of N-NVs did not induce pathological changes in mice (figs. S16 to S18). These findings demonstrated that the iSEND has no obvious side effects on experimental animals.

### The nanoDEX attenuated cytokine storm in an acute pneumonia mouse model and suppressed bone density reduction in an osteoporosis rat model

**Fig. 4.**
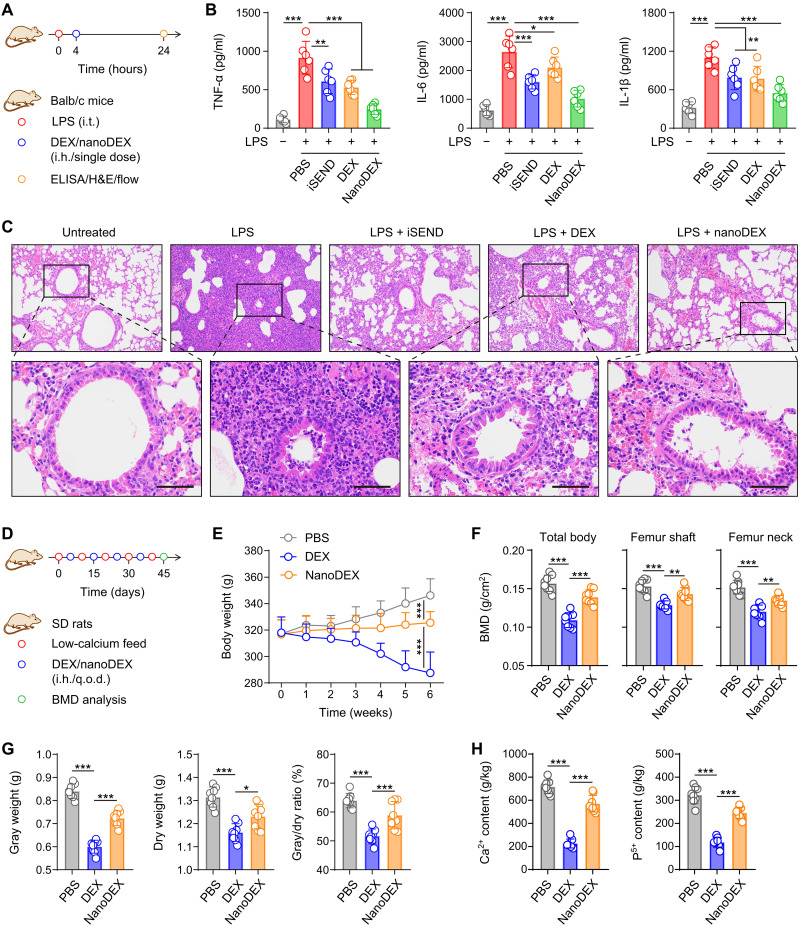
The nanoDEX attenuated lung injury in an acute pneumonia mouse model and suppressed bone density reduction in an osteoporosis rat model. (**A**) Schematic showing the treatment schedule in an acute pneumonia mouse model. (**B**) The cytokine productions in the lung homogenate after indicated treatments. (**C**) Hematoxylin and eosin (H&E)–stained lung tissues after indicated treatments. Scale bars, 50 μm. (**D**) Schematic showing the treatment schedule in a GIO rat model. (**E**) Body weight change curves after indicated treatments. (**F**) Bone mineral density (BMD) analysis in total body, femur shaft, and femur neck. (**G**) Bone gray weight, dry weight, and gray/dry ratio analysis after indicated treatments. (**H**) Ca^2+^ and P^5+^ content in the bone after indicated treatments. All data are presented as means ± SD [*n* = 6 (B) and *n* = 8 (E to H)]. Statistical significance was calculated via ordinary one-way ANOVA with Dunnett’s test (B and F to H) or two-way ANOVA with Tukey’s test (E). *P < 0.05, **P < 0.01, and ***P < 0.001. i.t., intratracheal; ELISA, enzyme-linked immunosorbent assay; q.o.d., every other day.

The nanoDEX protects against COVID-19 cytokine storm through a two-step strategy: cytokine down-regulation by DEX in the first step followed by cytokine neutralization by the iSEND in the second step. To test the effect of nanoDEX on the inhibition of COVID-19 cytokine storm, we first tested the formula in an LPS-induced acute pneumonia mouse model. At 4 hours after the LPS treatment, the mice received inhalation delivery of nanoDEX, and at 24 hours after the LPS challenge, all mice were euthanized, and lung tissues were collected for cytokine measurement (Fig. 4A). In the lung homogenate, the TNF-α, IL-6, and IL-1β levels were significantly up-regulated after the LPS challenge (Fig. 4B), demonstrating the inflammatory status in the lung. The nanoDEX significantly decreased the cytokine levels in the lung (Fig. 4B), which is attributed to efficient cytokine down-regulation by DEX synergized with cytokine neutralization by the iSEND. Moreover, the LPS treatment significantly improved the proportion of CD3^+^CD45^+^ T cells, CD14^+^CD45^+^ inflammatory infiltrating monocytes, and CD11b^low^F4/80^hi^ resident macrophages in the lungs (fig. S19), which play crucial roles in acute pneumonia (*43*). The proportion of these key immune cells was significantly lower in mice treated with nanoDEX than in those treated with DEX or iSEND alone (fig. S19), indicating effective suppression of immune disorder status. Furthermore, histological examination revealed that the nanoDEX significantly suppressed the LPS-induced severe lung injury characterized by alveolar cavity disappearance, alveolar wall incrassation, inflammatory cell infiltration, and vascular dilatation and congestion (Fig. 4C and table S1), indicating the potential of nanoDEX against the COVID-19–associated lung injury.

Despite its promising anti-inflammation effect, DEX, as a glucocorticoid, may lead to severe side effects such as femoral head necrosis (*16*, *17*). To investigate the safety of nanoDEX, we developed a rat model of glucocorticoid-induced osteoporosis (GIO) (*44*). The rats received low-calcium feed and inhalation management of DEX or nanoDEX every other day (q.o.d.) (Fig. 4D). We observed that the rats lost weight rapidly after the DEX treatment, whereas the rats treated with nanoDEX exhibited weight stabilization and gradually maintained their health after 4 weeks (Fig. 4E). All rats were euthanized for bone health analysis after a 6-week monitoring. Both in the femur shaft and neck, the bone mineral densities (BMDs) were significantly decreased after the DEX treatment; in contrast, inhalation delivery of DEX with iSEND suppressed DEX-induced BMD decrease (Fig. 4F). Moreover, we demonstrated that the DEX treatment caused the decrease of bone gray/dry weight and the loss of bone calcium/phosphorus contents (Fig. 4, G and H), demonstrating typical GIO status in rats. Notably, the nanoDEX treatment significantly protected rats from bone weight decrease and calcium/phosphorus loss (Fig. 4, G and H). These results suggested that inhalation delivery of nanoDEX significantly protected rats from DEX-induced side effects, which is attributed to reduced nonspecific distribution of DEX.

### The nanoDEX attenuated lung inflammation and injury in K18-hACE2 transgenic mice challenged with SARS-CoV-2

**Fig. 5.**
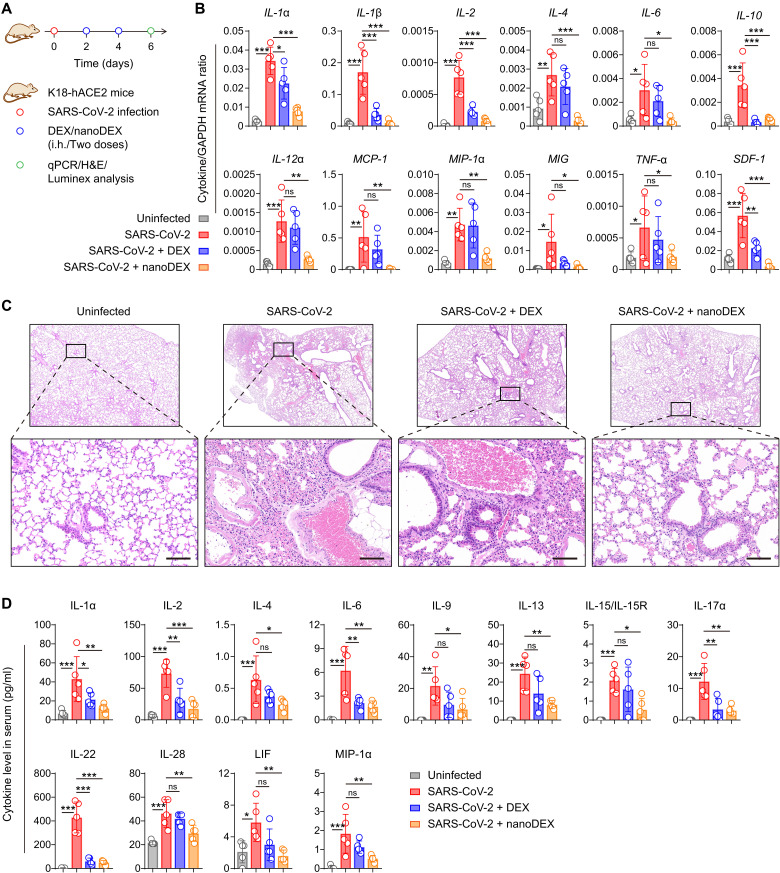
The nanoDEX attenuated lung inflammation and injury in K18-hACE2 transgenic mice challenged with SARS-CoV-2. (**A**) Schematic showing the treatment schedule in K18-hACE2 transgenic mice challenged with SARS-CoV-2. (**B**) Quantitative real-time polymerase chain reaction (qRT-PCR) analysis of cytokine gene expression in the lungs after indicated treatments. (**C**) H&E-stained lung tissues after indicated treatments. Scale bars, 100 μm. (**D**) Multiplex immunoassay analysis of cytokine/chemokine levels in the serum after indicated treatments. All data are presented as means ± SD (*n* = 5). Statistical significance was calculated via ordinary one-way ANOVA with Dunnett's test. *P < 0.05, **P < 0.01, and ***P < 0.001.

The K18-hACE2 transgenic mice expressing human SARS-CoV-2 receptor [i.e., angiotensin-converting enzyme 2 (hACE2)] under a cytokeratin 18 promoter (K18) are susceptible to SARS-CoV-2 infection, resulting in a dose-dependent lung inflammation disease course (*45*). We thus selected the K18-hACE2 mice to test anti–COVID-19 efficacy of the nanoDEX. At days 2 and 4 after the challenge with live SARS-CoV-2 by intranasal route (*46*, *47*), the K18-hACE2 mice received noninvasive inhalation delivery of DEX or nanoDEX (*48*). At day 6 after the challenge, all mice were euthanized, and blood samples and lung tissues were collected for cytokine/chemokine measurement and histological examination (Fig. 5A). At genetic level, SARS-CoV-2 infection induced the up-regulation of most of the detected cytokines/chemokines in the lung including IL-1α, IL-1β, IL-2, IL-4, IL-6, IL-10, IL-12α, monocyte chemoattractant protein-1 (MCP-1), macrophage inflammatory protein-1α (MIP-1α), monokine induced by IFN-γ (MIG), TNF-α, and stromal cell–derived factor-1 (SDF-1) (Fig. 5B and fig. S20A) but had little or down-regulation effect on specific cytokines/chemokines (fig. S20B), suggesting the COVID-19 cytokine storm in the lung (*8*, *49*). The nanoDEX treatment effectively inhibited SARS-CoV-2 infection–induced cytokine/chemokine up-regulation in the lung as compared to the DEX treatment (Fig. 5B), which is probably attributed to enhanced delivery of DEX into lung tissues and synergetic cytokine neutralization by iSEND. Furthermore, histological examination of lung tissues from SARS-CoV-2–infected mice showed a variety of inflammatory lesions including inflammatory cell infiltration, alveolar edema, alveolar cavity disappearance, and alveolar wall incrassation (Fig. 5C and table S2). Notably, inhalation delivery of nanoDEX significantly suppressed the lung injury (Fig. 5C), indicating the potential of this inhalable nanoDEX for the COVID-19–associated immune disorder and lung injury. Consistent with local inflammation findings, multiplex immunoassay analysis of cytokines/chemokines in the serum confirmed that the nanoDEX effectively suppressed systemic inflammation induced by the SARS-CoV-2 infection (Fig. 5D and fig. S21). In addition, we observed that the mice lost weight rapidly after the DEX treatment, whereas the mice treated with nanoDEX exhibited better weight stabilization (fig. S22), further suggesting the biosafety of our inhalable nanoDEX.

### A 10-fold lower dose of nanoDEX attenuated lung inflammation and injury in rhesus macaques challenged with SARS-CoV-2

**Fig. 6.**
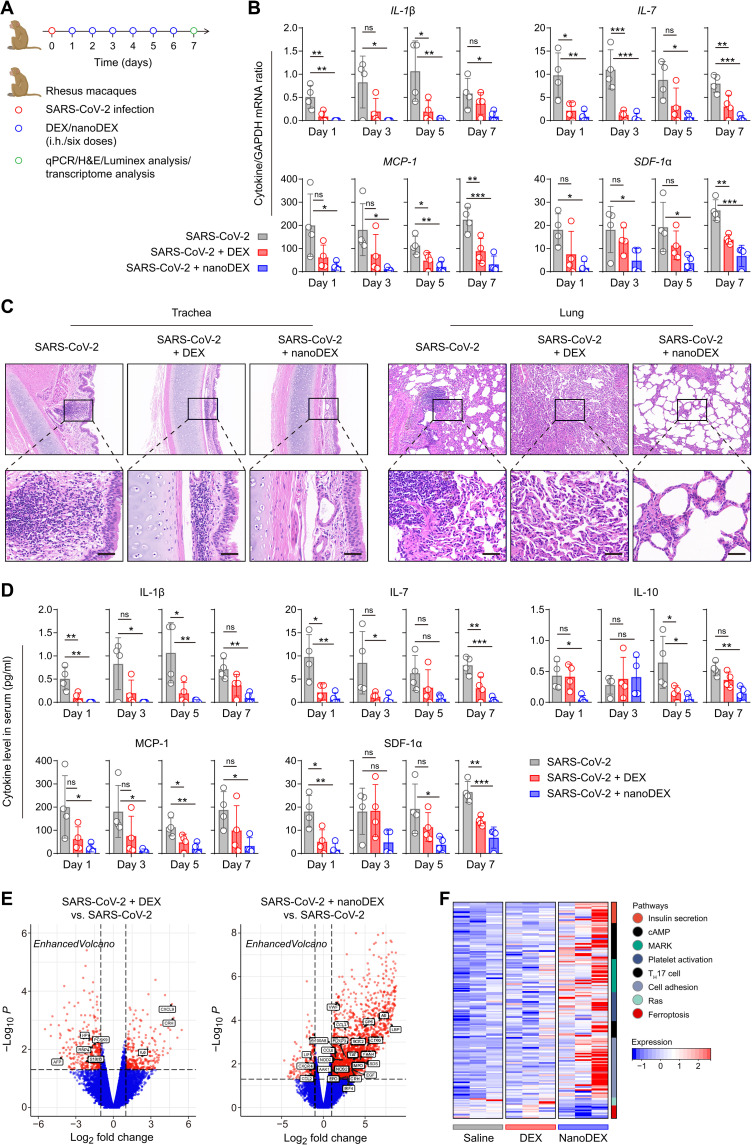
The nanoDEX attenuated lung inflammation and injury in rhesus macaques challenged with SARS-CoV-2. (**A**) Schematic showing the treatment schedule in rhesus macaques challenged with SARS-CoV-2. (**B**) qRT-PCR analysis of cytokine gene expression in the serum after inhalation delivery of DEX (0.1 mg/kg) or nanoDEX (0.01 mg/kg). (**C**) H&E-stained trachea and lung tissues after indicated treatments. Scale bars, 50 μm. (**D**) Multiplex immunoassay analysis of cytokine/chemokine levels in the serum after indicated treatments. (**E**) Volcano of differentially expressed genes in lungs among different groups. The genes associated with inflammation were marked by black boxes. (**F**) Heatmap of genes associated with inflammation- and injury-related pathways in different groups. All data are presented as means ± SD (*n* = 4). Statistical significance was calculated via ordinary one-way ANOVA with Dunnett’s test. *P < 0.05, **P < 0.01, and ***P < 0.001. cAMP, cyclic adenosine monophosphate; MARK, microtubule affinity-regulating kinase; T_H_17, T helper cell 17.

Furthermore, we used rhesus macaques to test anti–COVID-19 efficacy of our inhalable nanoDEX. Considering that the nanoDEX had about 14-fold improved accumulation in inflamed lungs based on mouse models, the macaques were infected live SARS-CoV-2 and then received inhalation delivery of DEX (0.1 mg/kg) or a 10-fold lower dose of nanoDEX (0.01 mg/kg) once daily. At day 7 after the challenge, all macaques were euthanized, and blood and tissues were collected for measurement and analysis (Fig. 6A). At genetic level, SARS-CoV-2 infection induced the up-regulation of most of the detected cytokines in the serum (Fig. 6B and figs. S23 to S27), suggesting the COVID-19 cytokine storm in macaques (*50*). The nanoDEX treatment effectively inhibited SARS-CoV-2 infection–induced cytokine/chemokine up-regulation as compared to the DEX treatment (Fig. 6B), which is probably attributed to enhanced delivery of DEX into lung tissues and synergetic cytokine neutralization by the iSEND. Furthermore, histological examination of trachea and lung tissues from SARS-CoV-2–infected macaques showed a variety of inflammatory lesions including inflammatory cell infiltration, tracheal mucosal cilia exfoliation, and alveolar structure destruction (Fig. 6C and table S3). Notably, inhalation delivery of nanoDEX significantly suppressed the lung injury (Fig. 6C), suggesting the great potential of this inhalable nanoDEX for the COVID-19–associated immune disorder and lung injury.

Consistent with the results at genetic level, multiplex immunoassay analysis of cytokines/chemokines in the serum confirmed that the nanoDEX effectively suppressed systemic inflammation induced by the SARS-CoV-2 infection (Fig. 6D and figs. S28 to S32). Moreover, transcriptome results showed that most of the genes involved in inflammation and injury repair were up-regulated in the nanoDEX group but not in the DEX group (Fig. 6E and fig. S33A). Consistent with the volcano findings, the Kyoto Encyclopedia of Genes and Genomes (KEGG) analysis demonstrated more new coronavirus pathways involved in the nanoDEX group (fig. S33B). Furthermore, gene set analysis showed that, after the nanoDEX treatment, the gene sets associated with inflammatory repair began to be highly expressed, while the gene sets of the apoptosis pathway were significantly decreased (Fig. 6F), suggesting that the nanoDEX treatment actively participated in the process of inflammation and injury repair after COVID-19 infection. Overall, these results demonstrated that inhalation delivery of DEX with iSEND significantly improved the safety and efficacy of hormonotherapy for COVID-19.

## DISCUSSION

In summary, we have developed an inhalable nanoDEX for COVID-19. By taking advantage of inhalation delivery strategy and chemokine receptors on iSEND, the nanoDEX showed enhanced retention in inflamed lungs and improved targeting to activated macrophages and DCs, thus promoting the cytokine down-regulation effects of DEX and suppressing inflammatory cell infiltration and lung injury caused by the SARS-CoV-2 infection. Moreover, depending on cytokine receptors on iSEND, the nanoDEX neutralized broad-spectrum cytokines. The synchronous down-regulation and neutralization of cytokines enable efficient protection against the COVID-19 cytokine storm. Different from free DEX, the nanoDEX down-regulated certain SARS-CoV-2 infection–induced genes, which may be related to the involvement of iSEND. The iSEND showed superior in vivo safety, and inhalation delivery of nanoDEX effectively attenuated DEX-induced osteoporosis. Emerging biomaterials promise a variety of synthetic nanoparticles including inorganic and organic ones for targeted delivery of DEX *(**16**)*, while the in vivo biocompatibility of these nanoparticles needs to be further investigated. The simplicity, safety, and robust inflammation inhibition make our nanoDEX an attractive candidate for clinical development.

DEX is a highly active antiedema and antifibrotic agent (*15*). Because pulmonary edema and fibrosis have recently emerged as major complications in the management of COVID-19 (*8*), the nanoDEX could further potentiate the antiedema and antifibrotic effects of DEX by increasing drug activity and availability in hyperactivated immunocytes of inflamed lungs (*16*). The SARS-CoV-2 infection involves a complex mixture of immunocytes including monocytes, macrophages, DCs, and T cells, which together regulate COVID-19 development (*5*). Thus, the NVs of these cells can also be used for DEX delivery and combined with other therapeutic agents for synergistic treatment of COVID-19. While the current design uses DEX, it is envisioned that this nanomedicine platform can be generalized to many other types of glucocorticoids, such as methylprednisolone and betamethasone. Meanwhile, similar to COVID-19, other infections, chronic diseases, and traumatic injuries are also marked by inflammatory responses that can cause tissue injury and even organ dysfunction (*37*). The nanoDEX developed here is probably able to be expanded as a broad-spectrum platform to address some of these diseases and can be expected to ultimately improve clinical outcomes.

In realizing clinical application of the nanoDEX, we should be alerted to its in vivo biocompatibility. Much work is needed for comprehensive investigation of its short- and long-term toxicity, but our small-scale pilot study, together with the safety data in preclinical studies involving EVs (*22*, *24*), could more or less clear the safety concerns of iSEND. Scalability is also an essential factor that deserves to be considered for clinical translation (*51*). Regarding the source materials of iSEND, ex vivo production of neutrophils by advanced bioprocesses can supply clinical grade iSEND (*37*). Meanwhile, rapid development has been made in producing cells with universal immune compatibility, which also ensures the source supply for iSEND (*52*). Looking toward the future, the iSEND may open an exciting new area of personalized medicine. The cells can be collected from patients and the iSEND can be engineered before infusing them back, which guarantee the maximization of immune tolerance (*7*, *37*, *40*, *53*). Although further studies and optimizations are necessary, the nanoDEX represents a significant technological advancement with the potential to expand the therapeutic armamentarium for COVID-19 and other inflammatory diseases.

## MATERIALS AND METHODS

### Study design

The objective of the study was to develop an inhalable DEX nanomedicine to improve COVID-19 treatment and complication management. The in vivo anti-inflammation effect was assessed in an acute pneumonia mouse model, the in vivo bone density reduction investigation was assessed in an osteoporosis rat model, and the in vivo anti–COVID-19 infection study was assessed in K18-hACE2 transgenic mice and rhesus macaques challenged with SARS-CoV-2. All infectious experiments involving live SARS-CoV-2 were performed in an approved Animal Biosafety Level 3 facility. Sample sizes were determined on the basis of previous studies and experimental experience. Animals were randomly assigned to groups on the basis of animal age and body weight. The investigators were not blinded to allocation during experiments and outcome assessment. All experiments were run at least in triplicate.

### Cells and animals

The murine cell lines of RAW 264.7 macrophage-like cells and DC2.4 DC-like cells were obtained from the American Type Culture Collection (ATCC) and cultured under the guidelines offered by the ATCC. The Institute of Cancer Research (ICR) mice (male, 6-week-old) and rattus norvegicus rats (female, 3-month-old) were both purchased from Hunan Silaike Jinda Laboratory Animal Co. Ltd. (China). The K18-hACE2 mice (14-week-old) were purchased from GemPharmatech Co. Ltd. (China). The rhesus macaques (male, 3- to 4-year-old) were purchased from Anhui Shengpeng Experimental Animal Technology Co. Ltd. (China). All animal care and experimental procedures were approved by the Institutional Animal Care and Use Committee of Shenzhen Bay Laboratory, Southern Medical University, and Chinese Academy of Agricultural Sciences in accordance with the guidelines for the protection of animal subjects.

### Neutrophil collection

Neutrophils were isolated from the bone marrow of mice or rhesus macaques following established protocols (*38*). First, fresh bone marrow was flushed from the bone with RPMI 1640 (Gibco, USA), filtered through a 70-μm filter, and centrifuged at 450*g* for 10 min. After successive resuspension in erythrocyte lysate (TianGen, China) and PBS (Gibco, USA), neutrophils were isolated from the mixture with a Percoll (Solarbio, China) gradient method. Briefly, the obtained cell pellets were added to Percoll working solution with a Percoll gradient of 78, 70, and 58% (v/v) in PBS. After centrifugation at 490*g* for 30 min, neutrophils were recovered from the 58 and 70% interface and cultured in RPMI 1640 containing 10% exosome-depleted fetal bovine serum (FBS) (System Biosciences, USA). The cell population purity (Ly6G^+^CD11b^+^) and cell apoptosis (Beyotime, China) were identified using a CytoFLEX flow cytometer (Beckman Coulter, USA) with CytoExpert software (Beckman Coulter, USA). The isolated neutrophils were stained with Wright-Giemsa (Solarbio, China) and observed under an optical microscope (IX71, Olympus, Japan). The morphology of neutrophils was also characterized using a transmission electron microscope (JEM-2010HT, JEOL, Japan). The TEM sample of ultrathin section of neutrophils was prepared by engineers (Medical Research Center for Structural Biology, School of Basic Medical Sciences, Wuhan University, China).

### The iSEND preparation

To obtain N-NVs, the isolated neutrophils were disrupted by a hypotonic lysing buffer and a Dounce homogenizer. The solution was then treated with deoxyribonuclease and ribonuclease (Invitrogen, USA) and then centrifuged at 3200*g* for 5 min. The enriched supernatants were further centrifuged at 20,000*g* for 30 min and 80,000*g* for 1.5 hours, respectively. After that, the pellets were collected, washed with PBS mixing protease inhibitor tablet (Roche, Switzerland) for three times, sonicated for 5 min, and finally extruded stepwise through 400-, 200-, and 100-nm nanopore polycarbonate membranes on a mini extruder (Avanti Polar Lipids, USA). The hydrodynamic diameter and zeta potential of N-NVs were characterized with a dynamic light scatter (Nano-Zen 3600, Malvern Instruments, UK). The morphology of N-NVs was also characterized by TEM. The TEM samples were negatively stained with uranyl acetate. The stability of N-NVs in PBS and 10% FBS was monitored for 2 weeks by DLS. As a control, R-NVs were also prepared with similar protocols. To prepare the cholesterol-engineered N-NVs (iSEND), 100 μg of N-NVs was mixed with 20 μg of cholesterol and sonicated for 20 min. The samples were then centrifuged at 15,000*g* for 15 min to remove N-NVs, and the concentrations of cholesterols in the supernatant were measured using the Cholesterol Quantitative Assay Kit (Abcam, China) according to the manufacturer’s instructions.

### SDS-PAGE and Western blotting

For SDS-PAGE, neutrophil lysates and N-NVs were added into the protein extraction buffer, and the protein contents were measured with a bicinchoninic acid kit (Sigma-Aldrich, USA). The samples were heated at 95°C for 5 min, and 20 μg of each sample was loaded into a 10% SDS–polyacrylamide gel. The samples were run at 120 V for 2 hours, and the gel was stained with Coomassie blue for 4 hours and then decolorated overnight before the observation. For Western blotting, the protein samples obtained from R-NVs and N-NVs were denatured and loaded into 10% SDS–polyacrylamide gel. The segregated proteins were then transferred onto polyvinylidene fluoride membranes; blocked with 5% (w/v) skimmed milk at 25°C for 1 hour; incubated with CCR2, CXCR2, CXCR4, IL-6R, IL-1βR, and TNF-αR primary antibodies (all from Abcam) at 4°C overnight; and then further incubated with horseradish peroxidase–conjugated secondary antibody (Thermo Fisher Scientific, USA), and the blots were developed using a West Pico PLUS chemiluminescent substrate kit (Thermo Fisher Scientific, USA). The stability of the cytokine/chemokine receptors in N-NVs over 14 days and the stability of the cytokine/chemokine receptors before and after DEX loading into N-NVs were also investigated by Western blotting.

### Cytokine binding quantification

To determine the cytokine binding capability of N-NVs, 100 μl of PBS containing different concentrations of R-NVs and N-NVs was mixed with 100 μl of PBS containing 1 ng of IL-6, IL-1β, and TNF-α and incubated for 2 hours at 37°C. The samples were then centrifuged at 15,000*g* for 15 min to remove the R-NVs and N-NVs, and the concentrations of IL-6, IL-1β, and TNF-α in the supernatant were measured by the corresponding enzyme-linked immunosorbent assay (ELISA) kit (eBioscience, USA) according to the manufacturer’s instructions. The ability of cytokine binding before and after DEX loading into N-NVs was also investigated.

### Drug loading and release

The loading of DEX (Sigma-Aldrich, USA) into N-NVs was achieved as follows: 80 μg of DEX was mixed with indicated amounts of N-NVs and sonicated for 20 min. DEX-N-NVs were centrifuged and repeatedly washed with PBS to remove free DEX. All the washing solutions were collected, and the concentration of DEX was measured using a high-performance liquid chromatograph (Alliance E2695, Walters, USA). The loading efficiency was calculated from the difference of the initial and left DEX in the supernatant. Side-by-side comparison of incubation, sonication, extrusion, and electroporation on DEX loading was further carried out. The release of DEX from N-NVs was investigated using the dialysis method. Briefly, DEX-N-NVs containing 100 μg of free DEX were loaded into dialysis bags with a molecular weight cutoff of 10 kDa (Millipore, USA). Dialysis bags were immersed in 50 ml of PBS and incubated for different times at 37°C with constant shaking at 70 rpm. At the indicated time points, 100 μl of the external medium was removed, and the same volume of fresh PBS was added. The concentration of DEX released into the bulk dialysis medium was determined by HPLC. As a control, DEX was also loaded into R-NVs with similar protocols.

### In vitro toxicity

Cell counting kit-8 (CCK-8) assay was used to evaluate the biocompatibility of N-NVs in vitro. Briefly, RAW 264.7 or DC2.4 cells were seeded in 96-well plates at a desired density and cultured for 12 hours. Then, different concentrations of R-NVs or N-NVs were added to the medium, and cells were incubated for 48 hours. Cells grown without any particles were used as a control. At the end of the incubation, CCK-8 (Sigma-Aldrich, USA) was added to test the cell viability according to the manufacturer’s instructions.

### In vitro targeting

RAW 264.7 or DC2.4 cells were seeded in 12-well tissue culture plates at 50% confluency and cultured overnight. Cell culture medium was changed, and LPS (100 ng/ml) from *Escherichia coli* (Sigma-Aldrich, USA) was added. After 4 hours of stimulation, cells were washed with PBS and fixed with 10% phosphate-buffered formalin (Thermo Fisher Scientific, USA) for 10 min and blocked with 1% BSA for 1 hour. DEX-R-NVs or DEX-N-NVs were labeled with 3,3′-dioctadecyloxacarbocyanine perchlorate (DiO; Thermo Fisher Scientific, USA), and the cells were then incubated with DiO-labeled DEX-R-NVs or DEX-N-NVs (0.2 mg/ml) in PBS at 4°C for 60 s. After incubation, cells were washed five times with ice-cold PBS, mounted with VECTASHIELD Antifade Mounting Medium with 4,6-diamidino-2-phenylindole (Vector Laboratories, USA), and imaged with confocal laser scanning microscopy (CLSM; LSM700, Zeiss, Germany). For flow cytometric analysis, cells were scraped and collected after PBS wash and then analyzed using a flow cytometer. Furthermore, DiO-labeled DEX-N-NVs (0.2 mg/ml) were mixed with IL-6, IL-1β, and TNF-α (10 ng/ml) and incubated for 2 hours at 37°C. The samples were further centrifuged at 15,000*g* for 15 min to remove residual cytokines, and the cells were then incubated with DiO-labeled DEX-N-NVs in PBS at 4°C for 60 s. After incubation, the cells were imaged with CLSM for florescence measurement.

### In vitro inflammation inhibition

RAW 264.7 cells were first cultured with LPS (100 ng/ml) for 1 hour, and R-NVs, N-NVs (equal concentration to R-NVs), DEX, or DEX-N-NVs (equal concentration to DEX and N-NVs) were added and incubated for additional 30 min. The supernatants were then collected and centrifuged at 15,000*g* for 15 min to remove the nanoparticles. TNF-α and IL-6 in the supernatant were measured by ELISA as described previously. The production of ROS was detected using a ROS assay kit (Sigma-Aldrich, USA). After the treatment of DEX-N-NVs, the cells were cultured with 20 mM 2′,7′-dichlorodihydrofluorescein diacetate (DCFH-DA; Sigma-Aldrich, USA) at 37°C for 30 min and then washed three times with a serum-free medium. The fluorescence signal intensity of DCFH-DA oxidatively transformed fluorescent dichlorofluorescein that was measured by flow cytometry, and the change of intracellular ROS level was measured.

### In vivo biodistribution

The ICR mice received intravenous or inhalation delivery of PBS containing 200 μg of DiO-labeled R-NVs or N-NVs with a commercially available portable nebulizer. At 24 hours after the treatment, the mice were euthanized, and all organs were carefully harvested and cryosectioned for further immunofluorescence analysis of the in vivo biodistribution of N-NVs after inhalation delivery. To test the inflammation targeting of N-NVs, an acute pneumonia model was developed according to previous report (*35*). The mice were anesthetized, placed in a supine posture, and then received inhalation delivery of PBS containing LPS (8 mg/kg). At 24 hours after the LPS challenge, the mice with acute pneumonia received inhalation delivery of PBS containing 200 μg of DiO-labeled R-NVs or N-NVs. At 24 hours after the treatment, the mice were euthanized, and all organs were carefully harvested for immunofluorescence analysis. The mice with acute pneumonia also received inhalation delivery of PBS containing 200 μg of DiO-labeled R-NVs or N-NVs. At 24, 48, and 72 hours after the treatment, the mice were euthanized, and all organs were carefully harvested for immunofluorescence analysis. The mice with acute pneumonia also received inhalation delivery of PBS containing the DiO-labeled N-NVs engineered with different concentrations of cholesterols. At 24 hours after the treatment, the mice were euthanized, and all organs were carefully harvested for immunofluorescence analysis. The mice with acute pneumonia also received intravenous or inhalation delivery of PBS containing DEX, DEX-R-NVs, DEX-N-NVs, or nanoDEX. At 24 hours after the treatment, the mice were euthanized, and all organs were carefully harvested for biodistribution analysis measured by HPLC. The mice with acute pneumonia received intravenous or inhalation delivery of PBS containing DiO-labeled nanoDEX (containing 200 μg of N-NVs). At 12, 24, and 48 hours after the treatment, the mice were euthanized, and lungs were carefully harvested and the fluorescence signal was measured by an IVIS imaging system (PerkinElmer).

### In vivo toxicity

To investigate the in vivo toxicity of iSEND, the ICR mice received inhalation delivery of PBS or PBS containing iSEND q.o.d. with a commercially available portable nebulizer. The body weight of mice was monitored and recorded over 15 days, and the mice were euthanized on day 15 after the first inhalation delivery. The blood samples were collected for complete blood test and serum biochemical examination with a blood biochemical autoanalyzer (7080, Hitachi, Japan). Major tissue samples were routinely made into sections described as above and stained with hematoxylin and eosin (H&E) for histological examination.

### In vivo inflammation inhibition in an acute pneumonia mouse model

The mice were anesthetized, placed in a supine posture, and then received inhalation delivery of PBS containing LPS (8 mg/kg) into the lung. At 4 hours after the challenge, the mice received inhalation delivery of PBS or PBS containing DEX (1 mg/kg) or equal amount of iSEND or nanoDEX. At 24 hours after the LPS challenge, all mice were euthanized, and the lungs were routinely collected. A part of lung tissues was weighed and homogenized with a lung homogenization medium. The IL-6, IL-1β, and TNF-α in the lung tissue homogenate were determined by the corresponding ELISA kit (eBioscience, USA). The other part of lung tissues was fixed in 4% neutral buffered formalin, processed into paraffin, and sectioned at 4 μm. The remaining lung tissues were used for flow cytometry.

### Flow cytometry

For flow cytometric analysis, single-cell suspensions of lung tissues were prepared using the lung dissociation kit (Miltenyi Biotech, China) according to the manufacturer’s instructions. Briefly, the lung tissues were perfused and dissected into single lobes. The resulting lobes were washed with PBS in a petri dish, placed in a C tube containing the enzyme mix to cut into small pieces using gentleMACS tissue dissociators, digested in an incubator for 1 hour at 37°C, and then filtered with 70-μm cell strainers (Becton Dickinson, USA). The cells were stained with fluorescence-labeled antibodies: Live/Dead (AF700; Becton Dickinson, USA), CD45 (APC-Cy7, clone 30-F11; Becton Dickinson, USA), CD3 (BV510, clone 145-2C11; Becton Dickinson, USA), CD14 (APC, clone Sa14-2; Biolegend, USA), CD11b (BB515, clone M1/70; Becton Dickinson, USA), F4/80 (APC, clone BM8; Becton Dickinson, USA), and CD206 (PE, clone BM8; Becton Dickinson). The samples were run on a CytoFLEX flow cytometer (Beckman Coulter, USA) with CytoExpert software.

### In vivo suppression of bone density reduction in an osteoporosis rat model

The rats received low-calcium feed daily and inhalation delivery of DEX (1 mg/kg) or equal amount of nanoDEX q.o.d. for 45 days. The body weight of rats was monitored to indicate the total health. After 45-day monitoring, all rats were euthanized, and the femur tissues were collected for BMD measurement by a bone density analysis system (X-Ray DXA, Kubtec, USA). All bone tissues were weighted and heated in muffle furnace for 6 hours, and the gray weight of bone tissues was further determined. Furthermore, a small amount of burned bone tissues was added into 10 ml of perchloric acid–concentrated sulfuric acid mixture (v/v, 1:5). After high-temperature digestion for 4 hours, the digested solution was kept for later use. The calcium and phosphorus contents in the bone were determined by flame atomic absorption spectrophotometry and ultraviolet spectrophotometry, respectively.

### In vivo suppression of lung injury in K18-hACE2 mice challenged with SARS-CoV-2

The K18-hACE2 transgenic mice were intranasally inoculated with 1.5 × 10^3^ plaque-forming units (PFU) of SARS-CoV-2 (the BetaCoV/Beijing/IMEBJ08/2020 strain) (*46*). On days 2 and 4 postinfection (p.i.), the mice received inhalation delivery of PBS or PBS containing DEX (1 mg/kg) or equal amount of nanoDEX separately via the pulmonary delivery method (*48*). Body weight was monitored daily. The body weight was monitored daily. On day 6 p.i., the mice were euthanized, and lung tissues and serum samples were harvested for cytokine measurement and histopathology analysis. The harvested lung tissue was fixed in 10% buffered formalin solution, and fixed tissues were then embedded in paraffin and sectioned at a thickness of 3 μm. The sections were stained with H&E and finally observed under an optical microscope.

### In vivo suppression of lung injury in rhesus macaques challenged with SARS-CoV-2

The rhesus macaques were intranasally inoculated with 1.2 × 10^6^ PFU of SARS-CoV-2 (the IME-BJ01 strain). On days 1 to 6 p.i., the macaques received inhalation delivery of PBS or PBS containing DEX (0.1 mg/kg) or nanoDEX (0.01 mg/kg) once daily (*15*). On day 7 p.i., the macaques were euthanized, and major tissues and serum samples were harvested for analysis. The harvested trachea and lung tissues were fixed, embedded, sectioned, stained with H&E, and finally observed.

### Measurement of cytokines/chemokines in lungs and serum

The levels of cytokine/chemokine in lungs were measured by quantitative real-time polymerase chain reaction (qRT-PCR). Total RNA was isolated from lung tissues using a multisource RNA miniprep kit (Axygen, USA) and reverse transcribed to cDNAs using an iScript cDNA synthesis kit (Bio-Rad, USA). The target genomes were quantified with qRT-PCR using the iTaq Universal SYBR Green Supermix (Bio-Rad, USA). qRT-PCR was performed using a Bio-Rad CFX-96 touch real-time detection system (the primers are shown in tables S4 and S5). The level of inflammatory factors was normalized with glyceraldehyde-3-phosphate dehydrogenase. The levels of cytokine/chemokine in serum of mice were quantified using a commercial ProcartaPlex mouse cytokine/chemokine multiple panel 1A kit (Thermo Fisher Scientific, USA). A panel of cytokines and chemokines including ENA-78 (epithelial neutrophil-activating protein 78; CXCL5), GRO-α (growth-regulated oncogene; CXCL1), G-CSF (granulocyte colony-stimulating factor), IFN-γ, IL-1α, IL-1β, IL-2, IL-4, IL-6, IL-9, TNF-α, IL-13, IL-15/IL-15R, IL-17α, IL-22, IL-23, IL-27, IL-28, LIF (leukemia inhibitory factor), MCP-1 (CCL2), MCP-3 (CCL7), and MIP-1α (CCL3) were measured for samples from mice according to the manufacturer’s protocols. The levels of cytokine/chemokine in serum of macaques were quantified using a commercial ProcartaPlex NHP cytokine/chemokine/growth factor panel 37plex kit (Thermo Fisher Scientific, USA). A panel of cytokines and chemokines including BDNF (brain-derived neurotrophic factor), B lymphocyte chemokine (CXCL13), Eotaxin (CCL11), FGF-2 (fibroblast growth factor 2), G-CSF (CSF-3), GM-CSF (granulocyte-macrophage colony-stimulating factor), IFN-α, IFN-γ, IL-1β, IL-10, IL-12p70, IL-13, IL-15, IL-17A (CTLA-8), IL-18, IL-1RA, IL-2, IL-23, IL-4, IL-5, IL-6, IL-7, IL-8 (CXCL8), interferon Gamma induced protein 10 (CXCL10), I-TAC (CXCL11), MCP-1 (CCL2), MIG (CXCL9), MIP-1α, MIP-1β (CCL4), NGF-β (β-subunit of NGF), PDGF-BB, sCD40L, SCF, SDF-1α (CXCL12a), TNF-α, VEGF-A (vascular endothelial growth factor A), and VEGF-D was measured for samples from macaques according to the manufacturer’s protocols.

### RNA sequencing and bioinformatic analysis

Rhesus macaque lung homogenates were subjected to RNA sequencing. Total RNA was extracted using the TRIzol (Invitrogen, USA) according to the manufacturer’s protocol. RNA purity and quantification were evaluated using the NanoDrop 2000 spectrophotometer (Thermo Fisher Scientific, USA). RNA integrity was assessed using the Agilent 2100 Bioanalyzer (Agilent Technologies, USA). The libraries were constructed using the TruSeq Stranded mRNA LT Sample Prep Kit (Illumina, USA) according to the manufacturer’s instructions. The transcriptome sequencing and analysis were conducted by OE Biotech Co. Ltd. (China). The libraries were sequenced on an Illumina HiSeq X Ten platform, and 150–base pair paired-end reads were generated. Raw data (raw reads) of fastq format were first processed using Trimmomatic, and the low-quality reads were removed to obtain the clean reads. The clean reads were mapped to the mouse genome (Mus_musculus GRCm38.99) using HISAT2 (*54*). The fragments per kilobase million of each gene was calculated using Cufflinks, and the read counts of each gene were obtained by HTSeq-count (*55*). Differential expression analysis was performed using the “edgeR” in R version 4.2.0 (version 4.2.0; www.r-project.org/). *P* < 0.05 and fold change > 2 were set as the threshold for significantly differential expression. Hierarchical cluster analysis of differentially expressed genes (DEGs) was performed to demonstrate the expression pattern of genes in different groups and samples. Gene Ontology enrichment and KEGG pathway enrichment analysis of DEGs were performed using the R package “clusterprofiler.” Heatmaps of gene expression levels were constructed using the “pheatmap” package in R.

### Statistical analysis

All results are presented as means ± SD. Ordinary one-way analysis of variance (ANOVA) with Tukey’s test (or Dunnett’s test) or two-way ANOVA with Tukey’s test was used for multiple group comparisons. All statistical analyses were performed with Prism 8.0.1 software (GraphPad).
