# Supplementary material for: Inhalation delivery of dexamethasone with iSEND nanoparticles attenuates the COVID-19 cytokine storm in mice and nonhuman primates
Source: Sci Adv. 2023 Jun 14;9(24):eadg3277. doi: 10.1126/sciadv.adg3277 (PMC10266725; doi:10.1126/sciadv.adg3277)
Supplement: Supplementary file 1 — Figs. S1 to S33 Tables S1 to S5 [file sciadv.adg3277_sm.pdf]

Science Advances
AAAS

Supplementary Materials for

Inhalation delivery of dexamethasone with iSEND nanoparticles attenuates the COVID-19 cytokine storm in mice and nonhuman primates

Qian-Fang Meng *et al.*

Corresponding author: Xiao Li, skylee6226@163.com; Gong Cheng, gongcheng@mail.tsinghua.edu.cn; Xiaoyuan Chen, chen.shawn@nus.edu.sg; Lang Rao, lrao@szbl.ac.cn

Sci. Adv. **9**, eadg3277 (2023)
DOI: 10.1126/sciadv.adg3277

This PDF file includes:

Figs. S1 to S33
Tables S1 to S5

Supplementary Figures and Tables

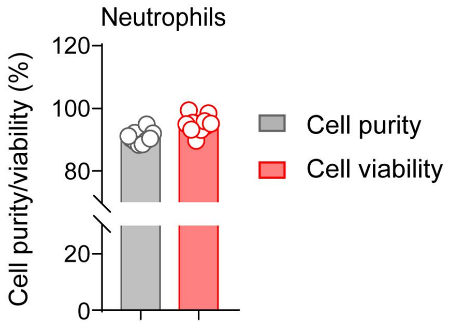

Supplementary Figure 1. Purity and viability of isolated neutrophils. All data are presented as mean ± S.D. (n = 10).

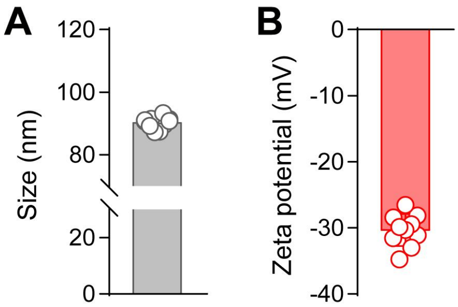

Supplementary Figure 2. (A) Size and (B) zeta potential of N-NVs in PBS. All data are presented as mean ± S.D. (n = 10).

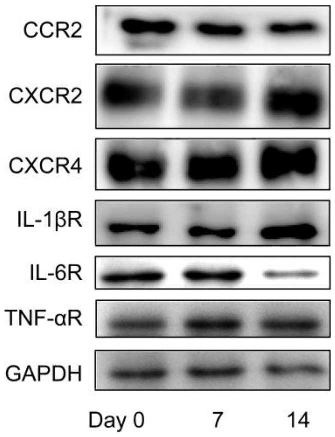

Supplementary Figure 3. Protein stability of N-NVs in PBS over 14 days.

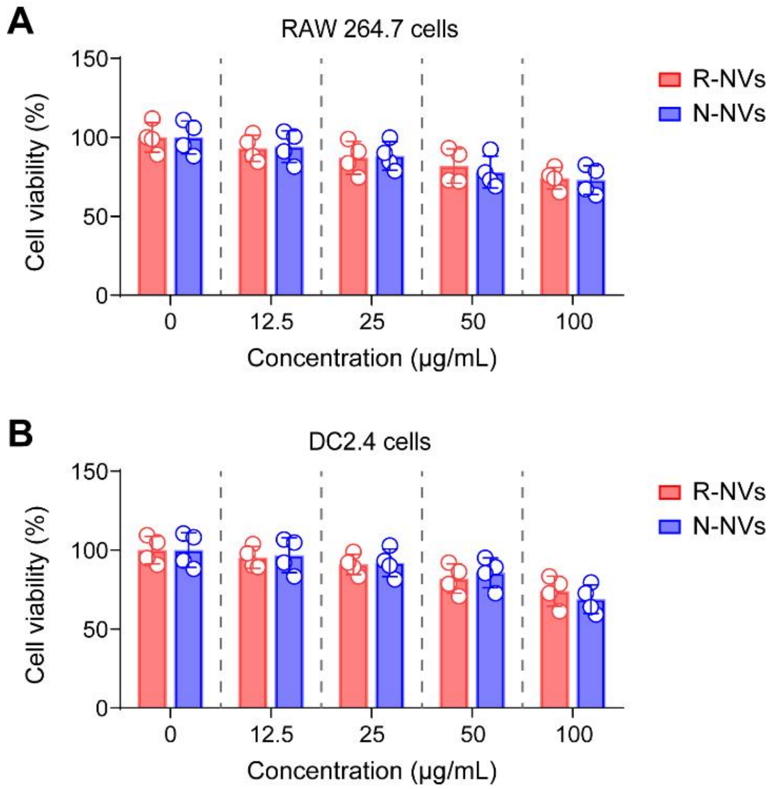

Supplementary Figure 4. (A) RAW 264.7 and (B) DC2.4 cell viability after incubation with indicated concentrations of R-NVs or N-NVs. All data are presented as mean ± S.D. ($n = 4$).

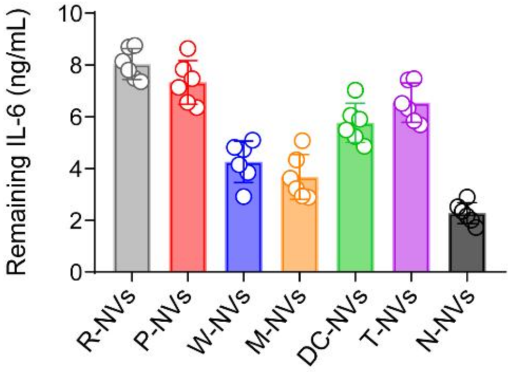

Supplementary Figure 5. Head-to-head comparison of the IL-6 binding capacity between different NVs derived from red blood cells (RBCs), platelets (PLTs), white blood cells (WBCs), macrophages, dendritic cells (DCs), T cells, and neutrophils. All data are presented as mean ± S.D. (n = 6).

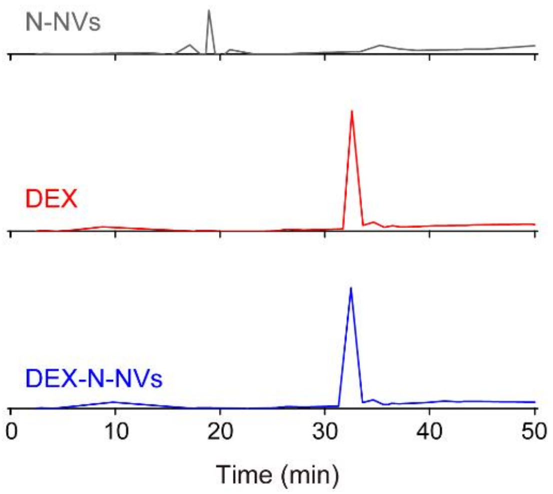

Supplementary Figure 6. HPLC analysis of N-NVs, DEX, and DEX-N-NVs. AU, arbitrary units.

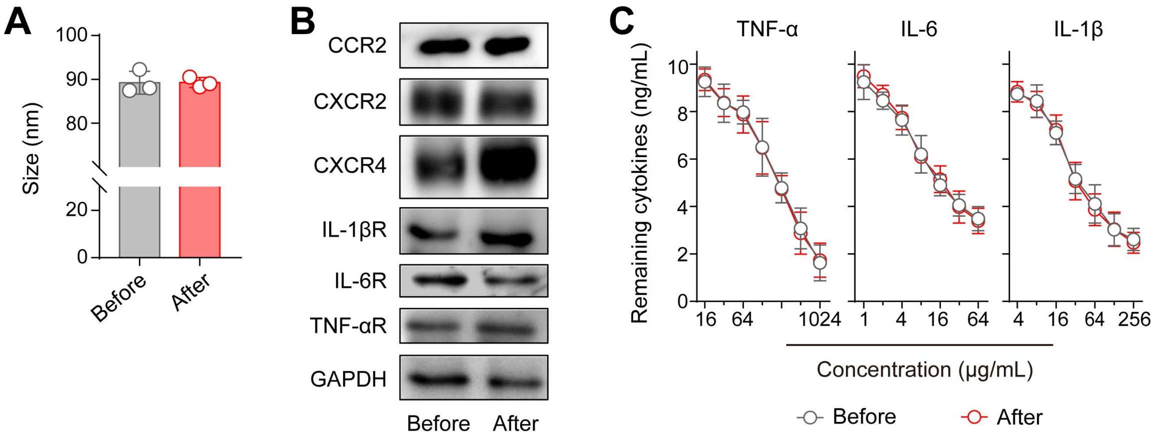

Supplementary Figure 7. Physicochemical properties of N-NVs before and after the sonication loading of DEX. (A) Size distribution of N-NVs before and after sonication. (B) Western blotting analysis of chemokine and cytokine receptors in N-NVs before and after sonication. (C) Binding capacity analysis of N-NVs with TNF-α, IL-6 and IL-1β before and after sonication. All data are presented as mean ± S.D. ($n = 3$).

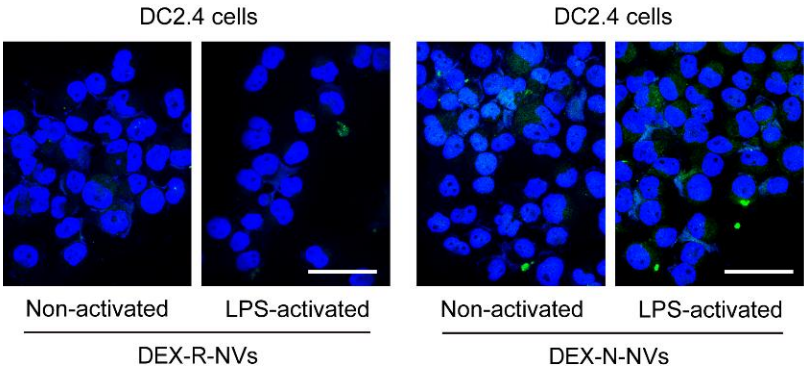

Supplementary Figure 8. Fluorescent images of non-activated or LPS-activated DC2.4 cells after incubation with DEX-R-NVs or DEX-N-NVs. Scale bar, 100 μm. DEX-R-NVs and DEX-N-NVs were labelled with DiO (green) before the incubation.

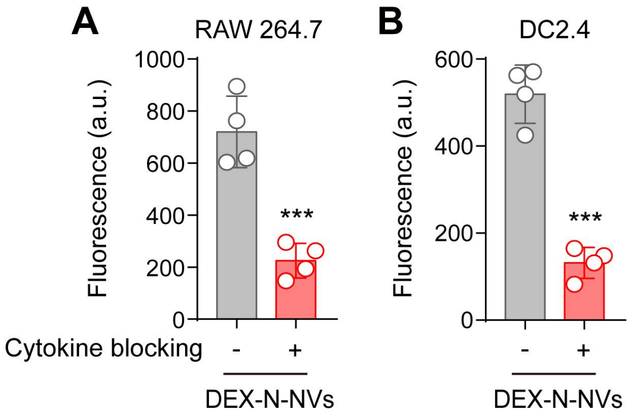

Supplementary Figure 9. Fluorescence intensity analysis of DiO (green) in (A) RAW 264.7 and (B) DC2.4 cells after incubation with DEX-N-NVs with or without cytokine blocking treatment. All data are presented as mean ± S.D. (*n* = 4).

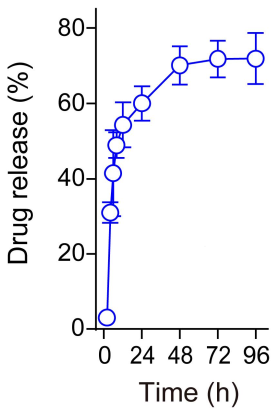

Supplementary Figure 10. Drug release profile of DEX-N-NVs after i.h. delivery. All data are presented as mean ± S.D. (n = 3).

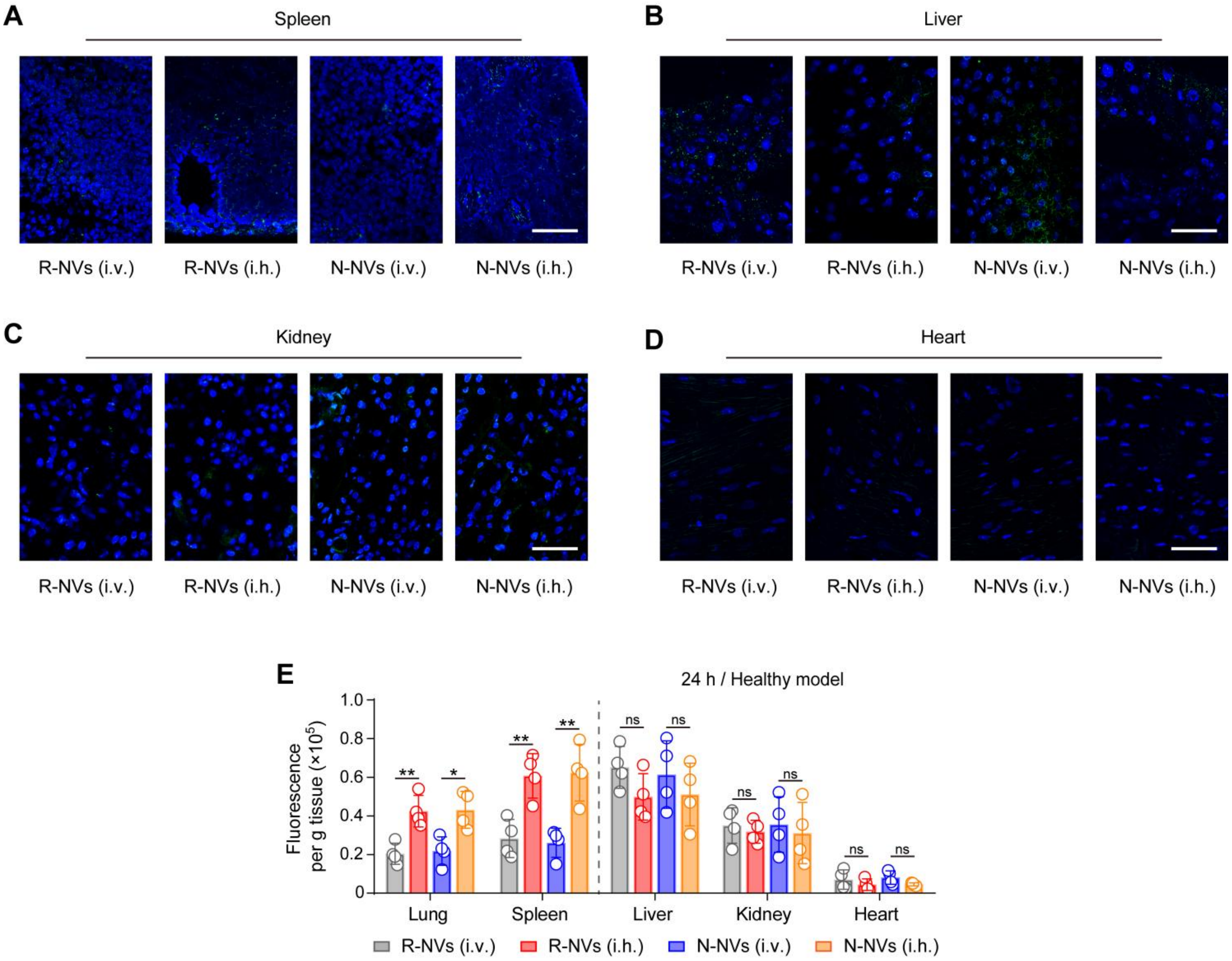

Supplementary Figure 11. Fluorescent images showing the accumulation of R-NVs or N-NVs in (A) spleen, (B) liver, (C) kidney and (D) heart after i.v. or i.h. delivery. Scale bars, 50 μm. R-NVs and N-NVs are labelled with DiO (green fluorescence) before the delivery. (E) Biodistribution of R-NVs or N-NVs in major organs at 24 h after i.v. or i.h. delivery in healthy mice. All data are presented as mean ± S.D. (n = 4). Statistical significance was calculated *via* ordinary one-way ANOVA with Tukey's test. *P < 0.05; **P < 0.01.

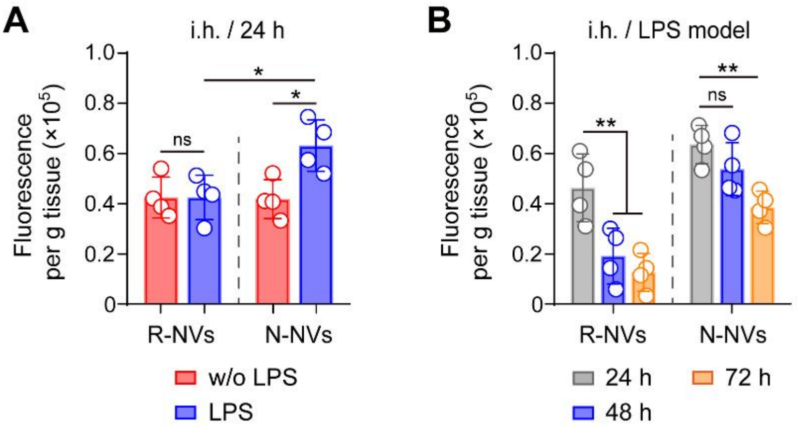

Supplementary Figure 12. (A) Lung accumulation of R-NVs or N-NVs at 24 h after i.h. delivery in healthy or LPS-infected mice. (B) Lung accumulation of R-NVs or N-NVs at different time points after i.h. delivery in LPS-infected mice. All data are presented as mean ± S.D. (n = 4).

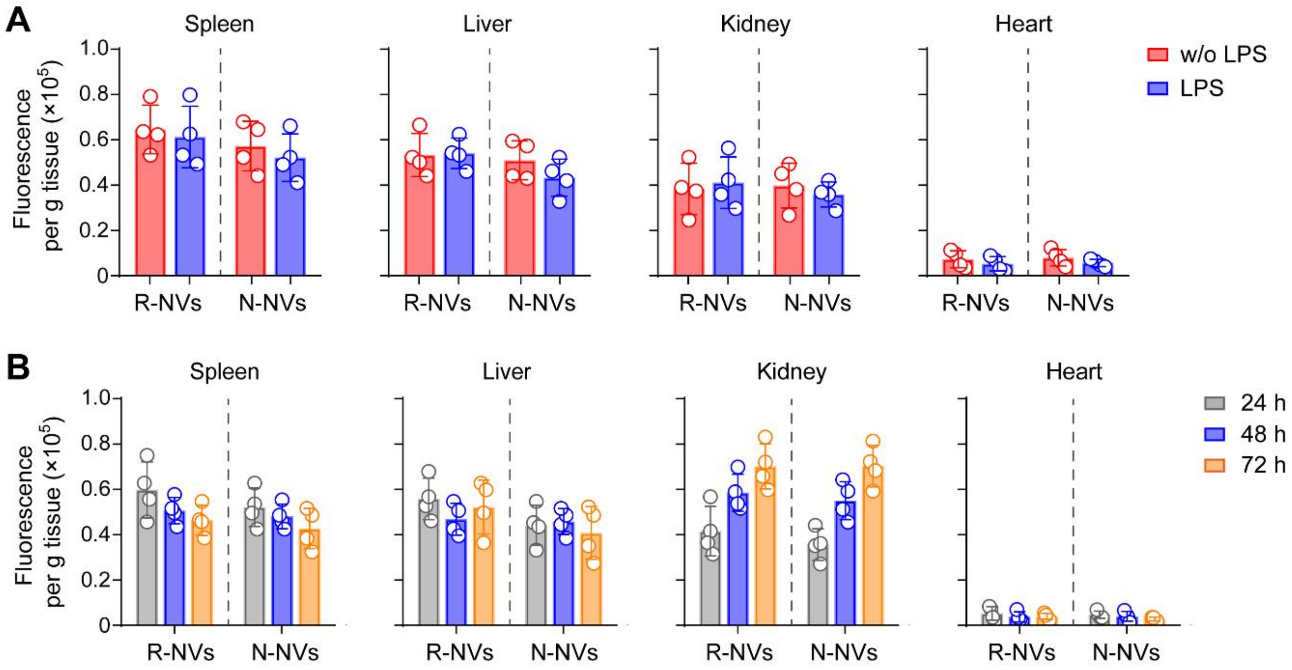

Supplementary Figure 13. (A) *In vivo* biodistribution of R-NVs or N-NVs in major organs at 24 h after i.v. or i.h. delivery in healthy or LPS-infected mice. (B) *In vivo* biodistribution of R-NVs or N-NVs in major organs at different time points after i.h. delivery in LPS-infected mice. All data are presented as mean ± S.D. (n = 4).

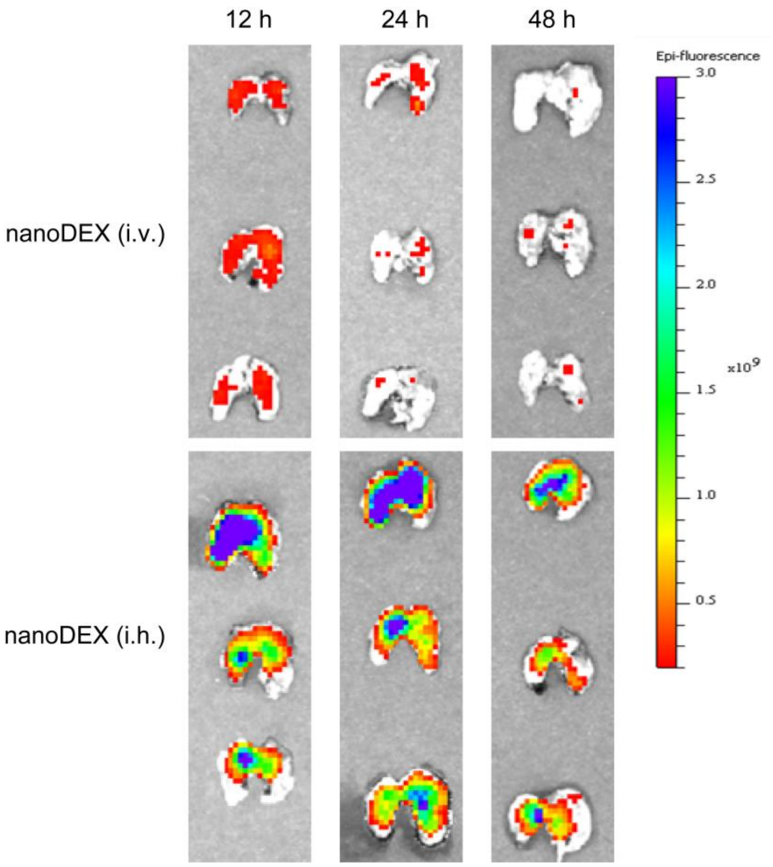

Supplementary Figure 14. Accumulation of nanoDEX in inflamed lungs at 12, 24, and 48 h after i.v. or i.h. delivery in LPS-infected mice. (n = 3).

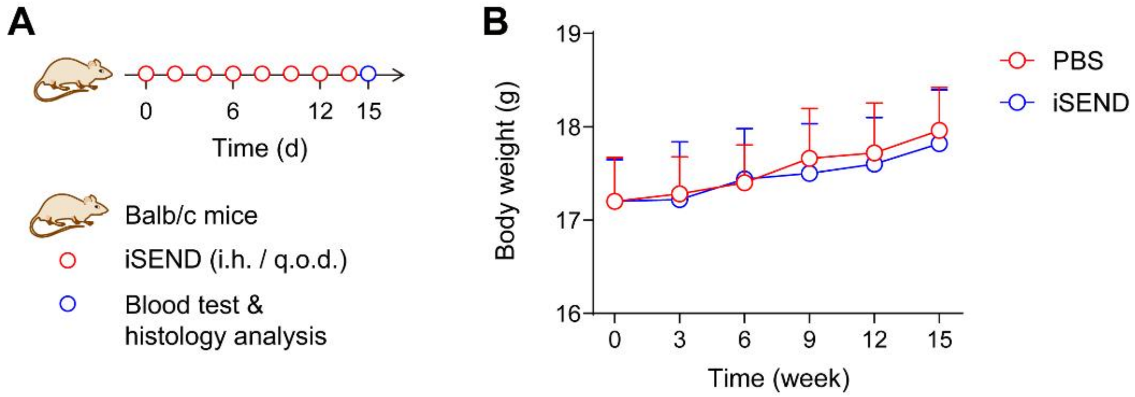

Supplementary Figure 15. *In vivo* toxicity of iSEND. (A) Schematic showing the treatment schedule. The mice received repeated i.h. delivery of PBS or PBS containing iSEND q.o.d. and blood samples and major organs were collected from mice 15 days after treatment for blood biochemistry, complete blood, and histology analysis. (B) Mice body weight change curves. All data are presented as mean ± S.D. (n = 5).

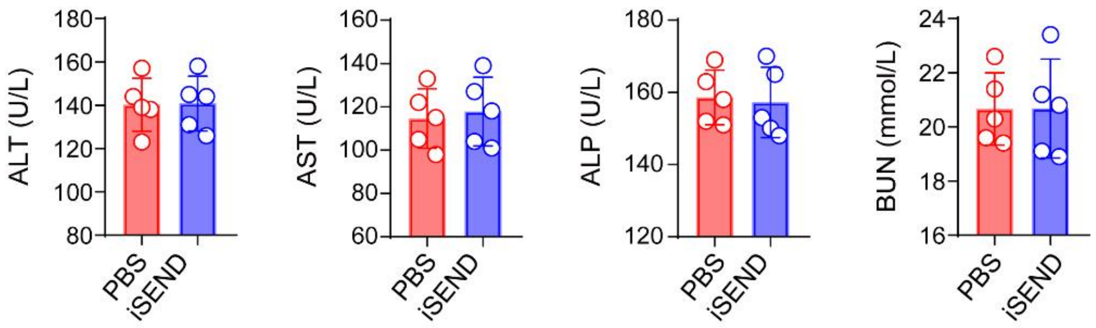

Supplementary Figure 16. Blood biochemistry analysis. ALT: alanine transaminase; AST: aspartate aminotransferase; ALP: alkaline phosphatase; BUN: blood urea nitrogen. All data are presented as mean ± S.D. ($n = 5$).

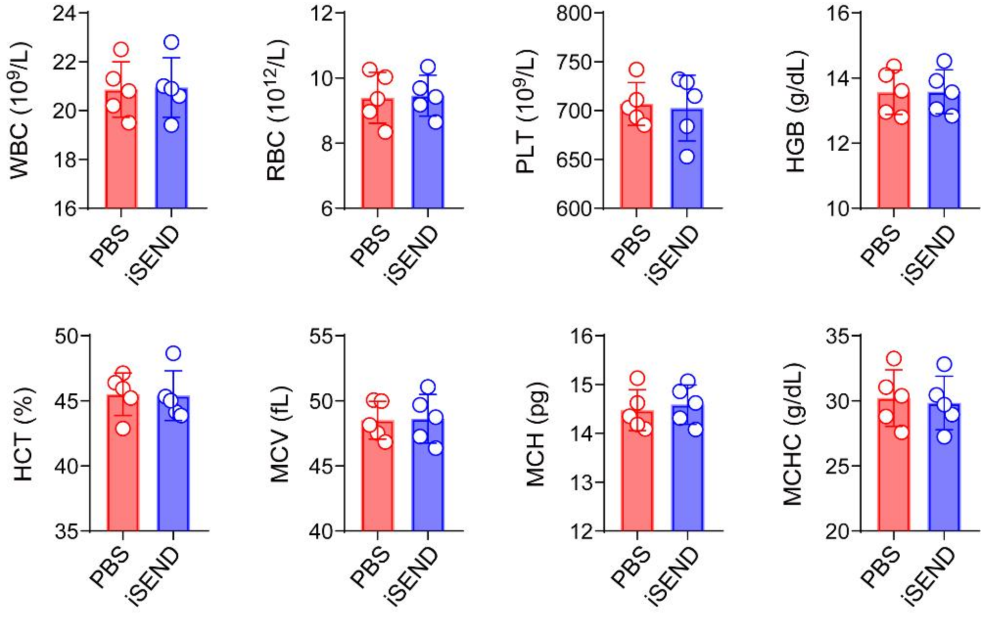

Supplementary Figure 17. Complete blood panel analysis. WBC: white blood cell; RBC: red blood cell; PLT: platelet; HGB: hemoglobin; HCT: hematocrit; MCV: mean corpuscular volume; MCH: mean corpuscular hemoglobin; MCHC: mean corpuscular hemoglobin concentration. All data are presented as mean ± S.D. ($n = 5$).

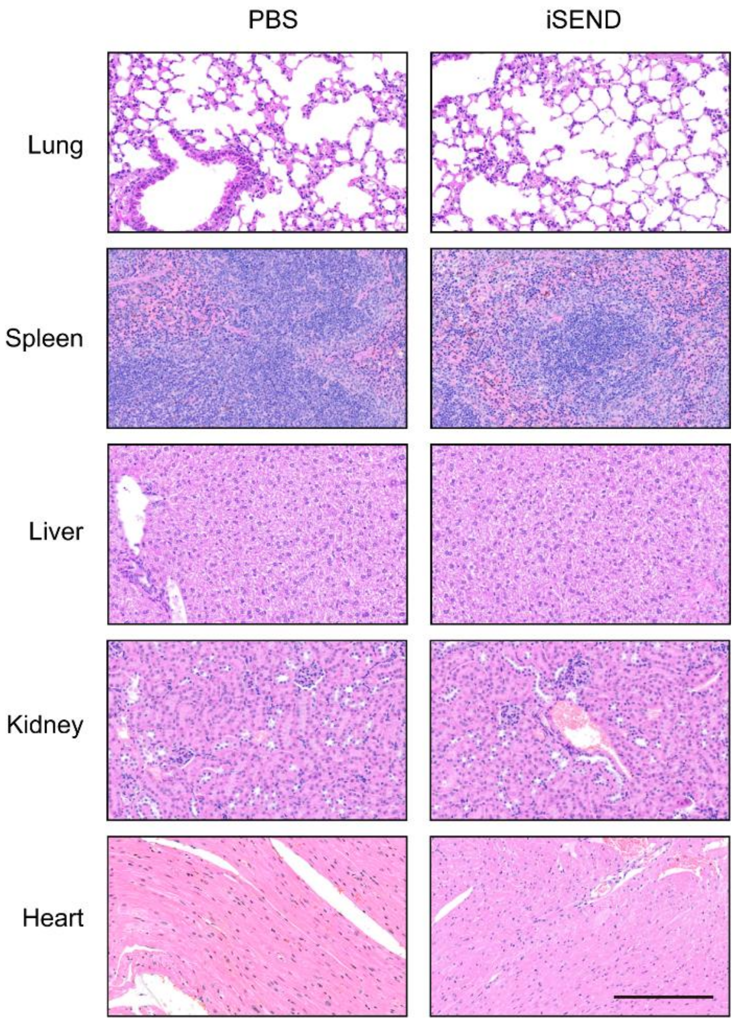

Supplementary Figure 18. H&E-stained slice images of major organs. Scale bar, 200 μm.

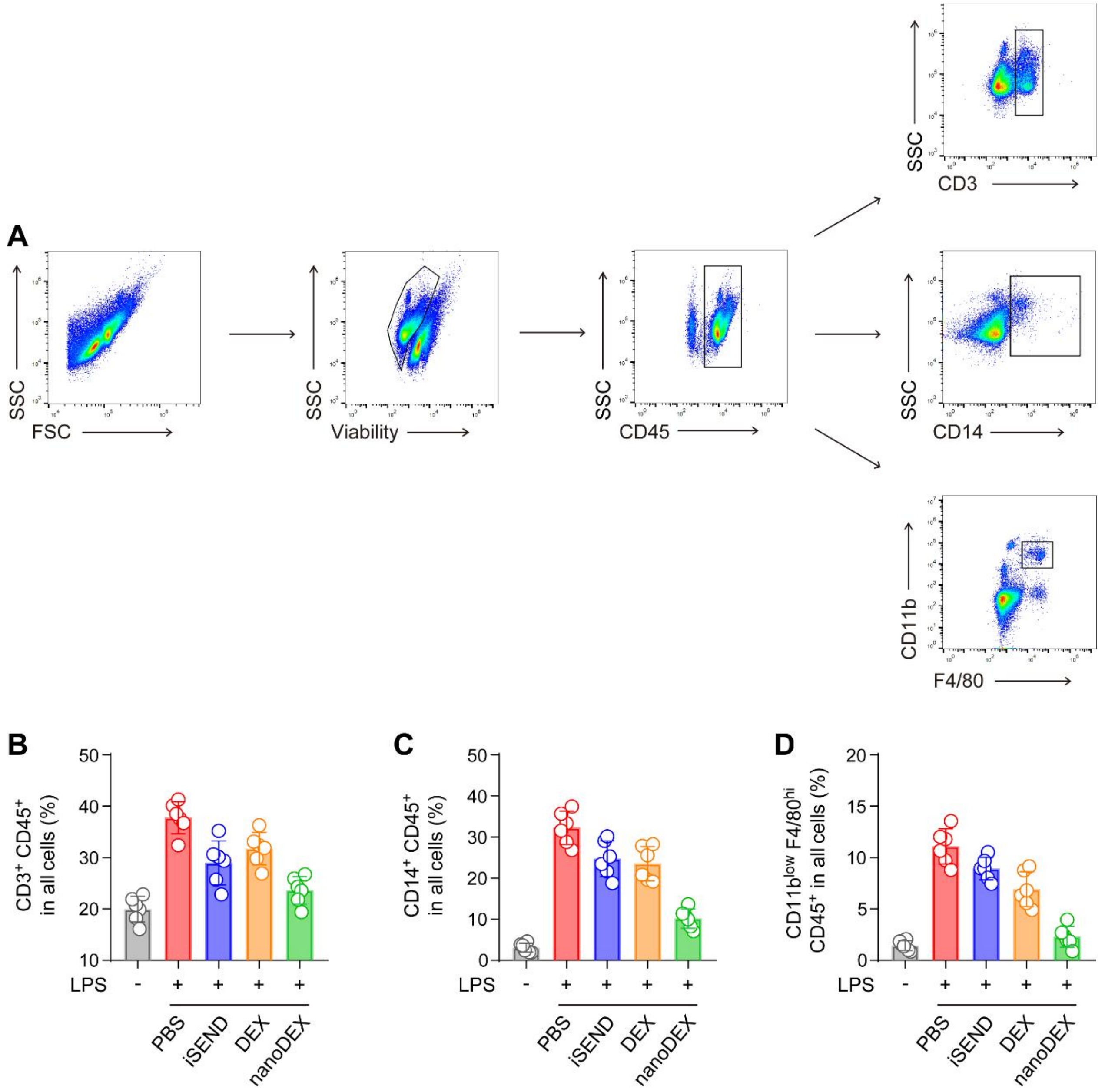

Supplementary Figure 19. Flow cytometry analysis of immunocytes in the lung homogenate after indicated treatments. (A) Graphically account for flow cytometry gating strategies. Flow cytometry analysis of (B) $CD3^{+}CD45^{+}$ T cells, (C) $CD14^{+}CD45^{+}$ inflammatory infiltrating monocytes/macrophages, and (D) $CD11b^{low}F4/80^{hi}$ resident macrophages. All data are presented as mean ± S.D. (n = 6).

Supplementary Table 1. Scoring of the inflammation observed in the H&E-stained lung sections in Figure 4C.

**Group**	Untreated	LPS	LPS + iSEND	LPS + DEX	LPS + nanoDEX
**Scoring**	0	3	2	2	1

The severity of inflammation observed in the H&E-stained lung/trachea sections was scored on a 0–3 scale defined as: 0 = no inflammatory response; 1 = mild inflammation with foci of inflammatory cells in bronchial or vascular wall and in alveolar septa; 2 = moderate inflammation with patchy inflammation or localized inflammation in walls of bronchi or blood vessels and alveolar septa, and less than one-third of the lung cross-sectional area is involved; 3 = severe inflammation with diffuse inflammatory cells in walls of bronchi or blood vessels and alveoli septa, and between one-third and two-thirds of the lung area is involved.

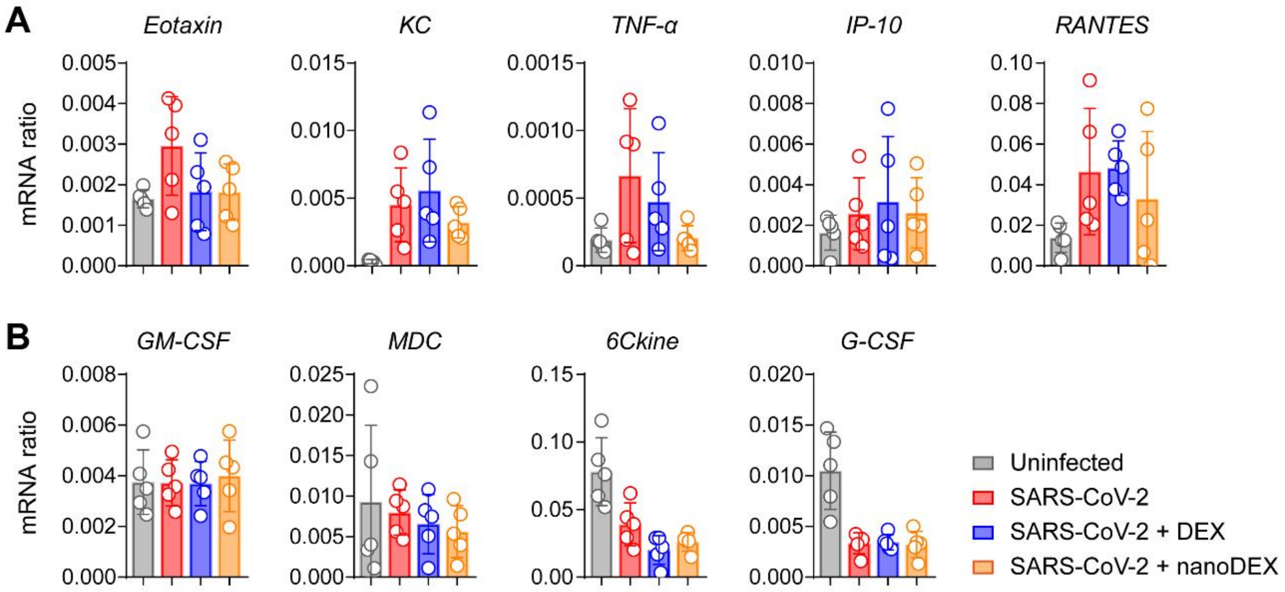

Supplementary Figure 20. qRT-PCR analysis of cytokine/chemokine expression in the lung after indicated treatments. (A) Eotaxin, KC, TNF-α, IP-10, and RANTES. (B) GM-CSF, MDC, 6Ckine and G-CSF. All data are presented as mean ± S.D. (n = 5).

Supplementary Table 2. Scoring of the inflammation observed in the H&E-stained lung sections in Figure 5C.

**Group**	Uninfected	SARS-CoV-2	SARS-CoV-2 + DEX	SARS-CoV-2 + nanoDEX
**Scoring**	0	2	1	1

The severity of inflammation observed in the H&E-stained lung/trachea sections was scored on a 0–3 scale defined as: 0 = no inflammatory response; 1 = mild inflammation with foci of inflammatory cells in bronchial or vascular wall and in alveolar septa; 2 = moderate inflammation with patchy inflammation or localized inflammation in walls of bronchi or blood vessels and alveolar septa, and less than one-third of the lung cross-sectional area is involved; 3 = severe inflammation with diffuse inflammatory cells in walls of bronchi or blood vessels and alveoli septa, and between one-third and two-thirds of the lung area is involved.

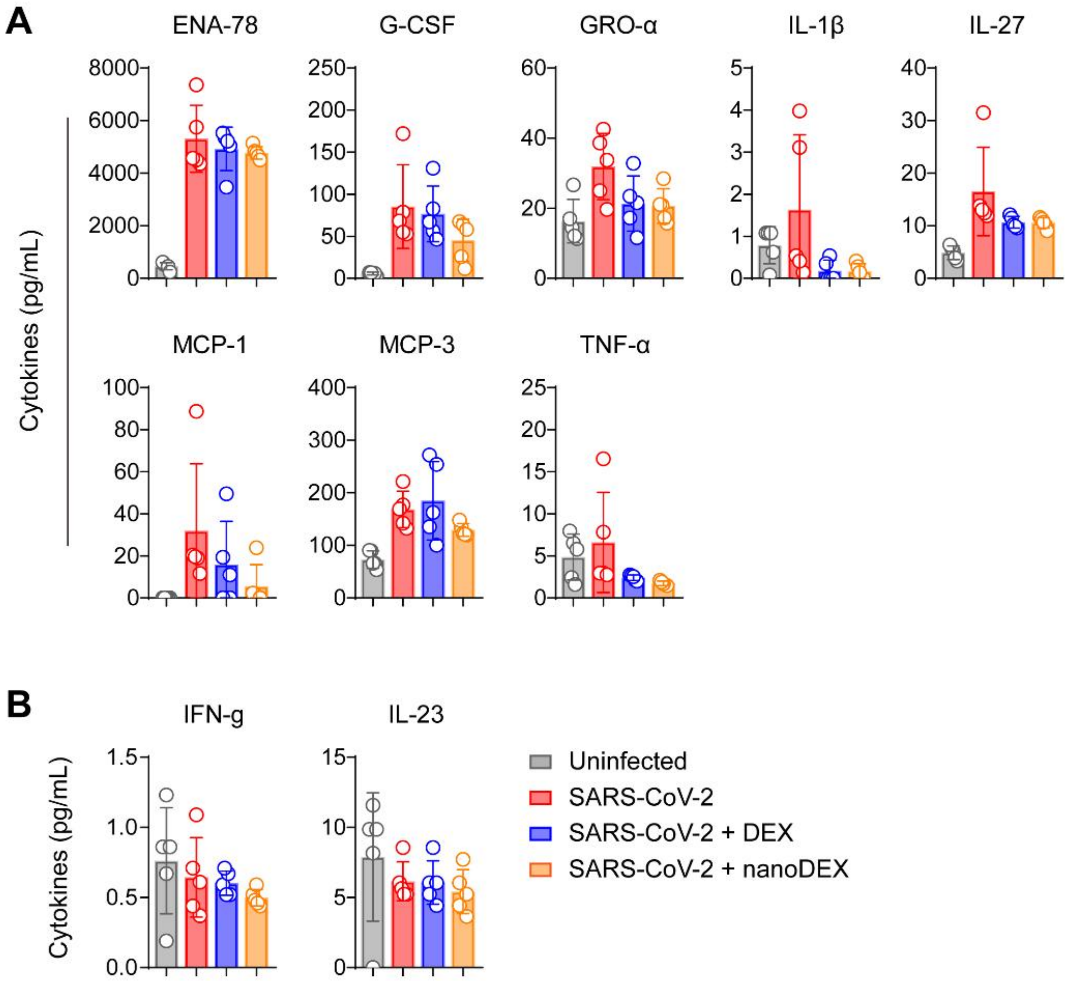

Supplementary Figure 21. Luminex analysis of cytokine expression in the serum after indicated treatments. (A) ENA-78, G-CSF, GRO-α, IL-1β, IL-27, MCP-3, MCP-1, and TNF-α. (B) IFN-g and IL-23. All data are presented as mean ± S.D. (n = 5).

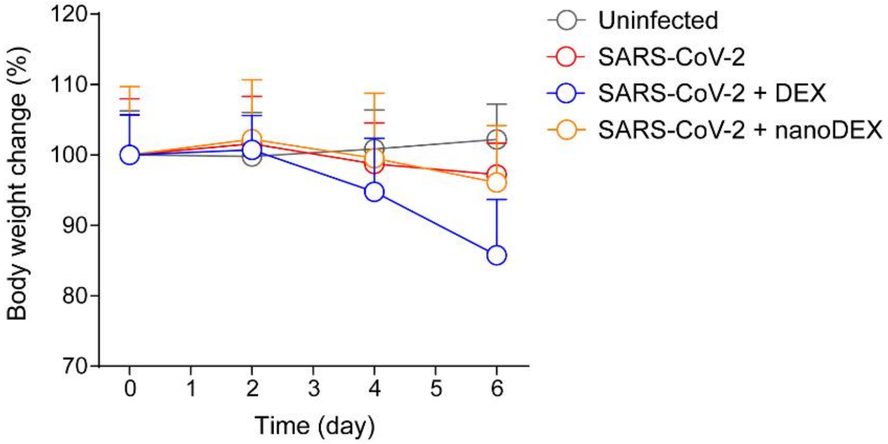

Supplementary Figure 22. Body weight change curves of K18-hACE2 transgenic mice challenged with live SARS-CoV-2 following indicated treatments. All data are presented as mean ± S.D. (*n* = 5).

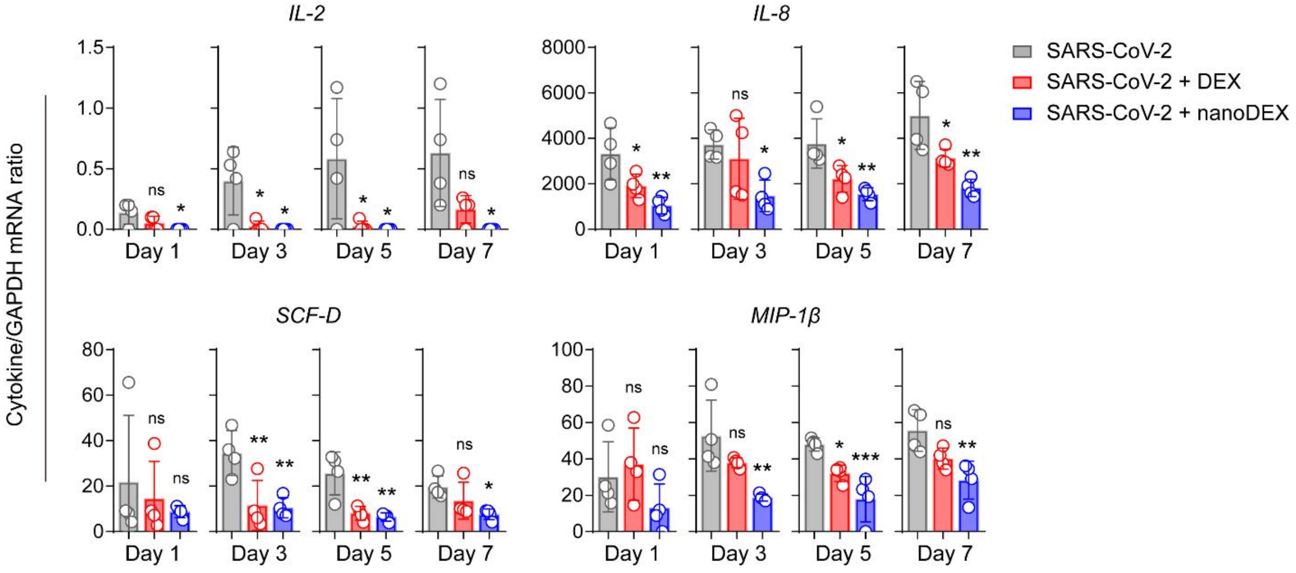

Supplementary Figure 23. qRT-PCR analysis of cytokine/chemokine expression in the serum after indicated treatments. All data are presented as mean ± S.D. (n = 4).

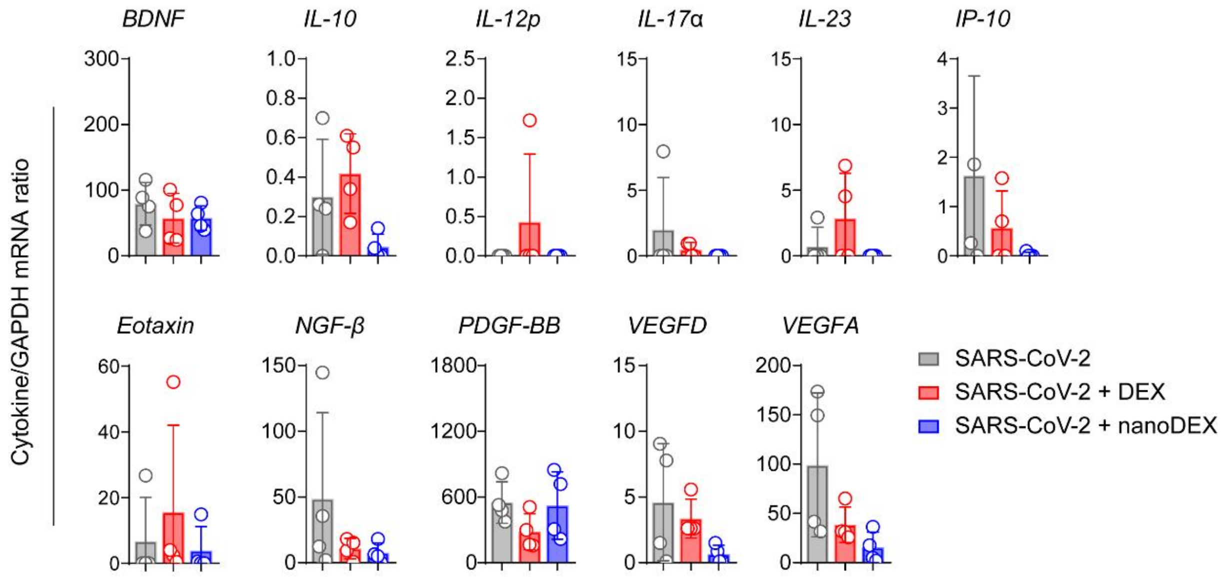

Supplementary Figure 24. qRT-PCR analysis of cytokine/chemokine expression in the serum at day 1 after indicated treatments. All data are presented as mean ± S.D. (n = 4).

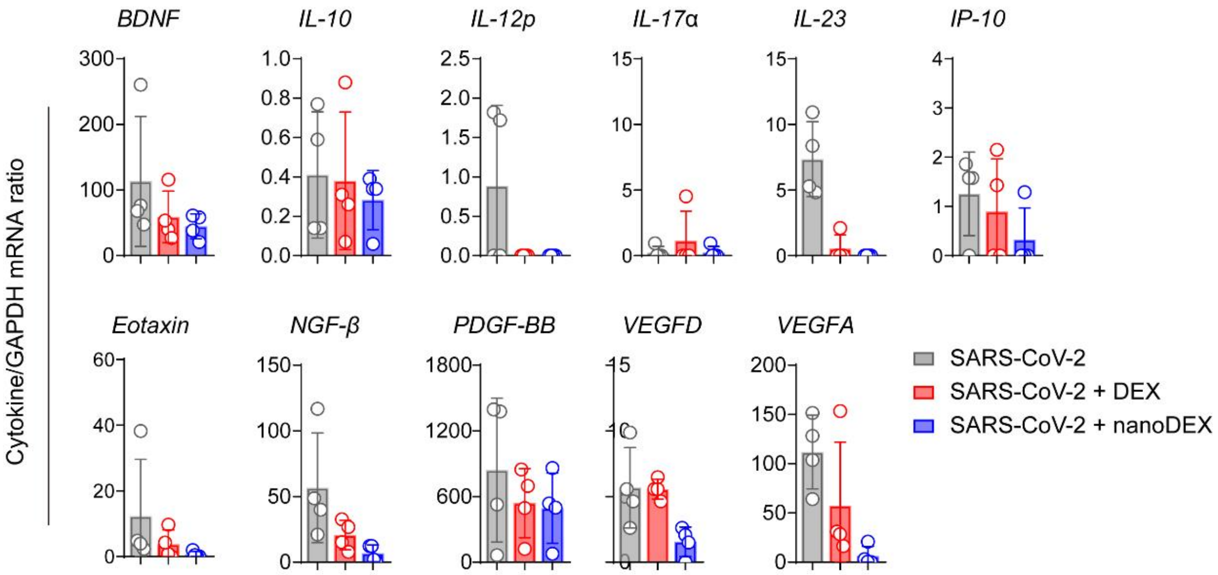

Supplementary Figure 25. qRT-PCR analysis of cytokine/chemokine expression in the serum at day 3 after indicated treatments. All data are presented as mean ± S.D. (*n* = 4).

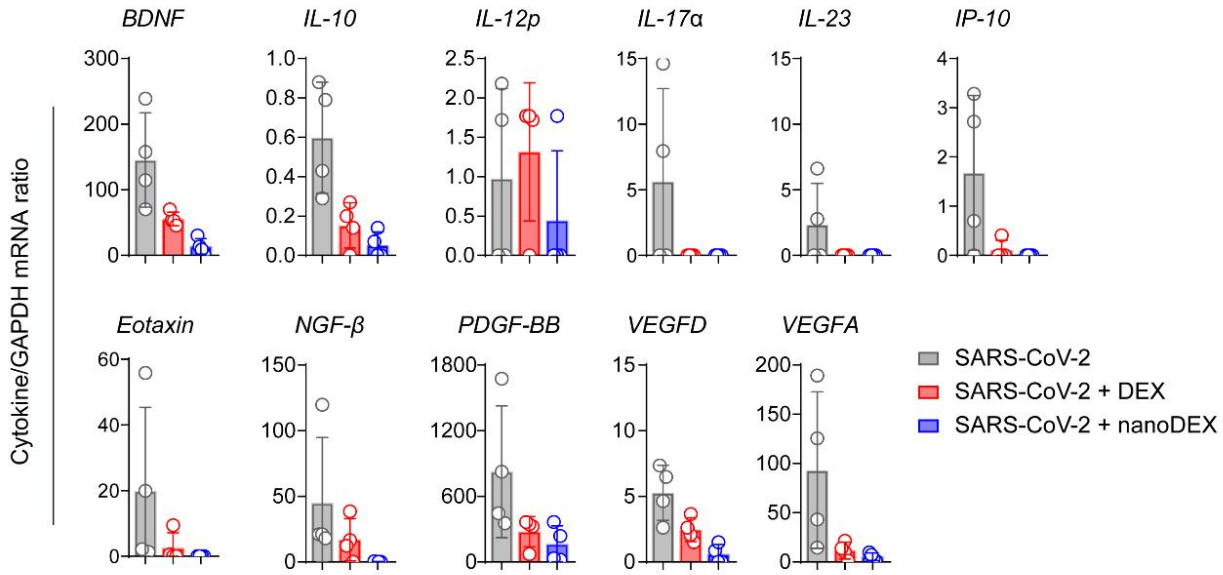

Supplementary Figure 26. qRT-PCR analysis of cytokine/chemokine expression in the serum at day 5 after indicated treatments. All data are presented as mean ± S.D. (n = 4).

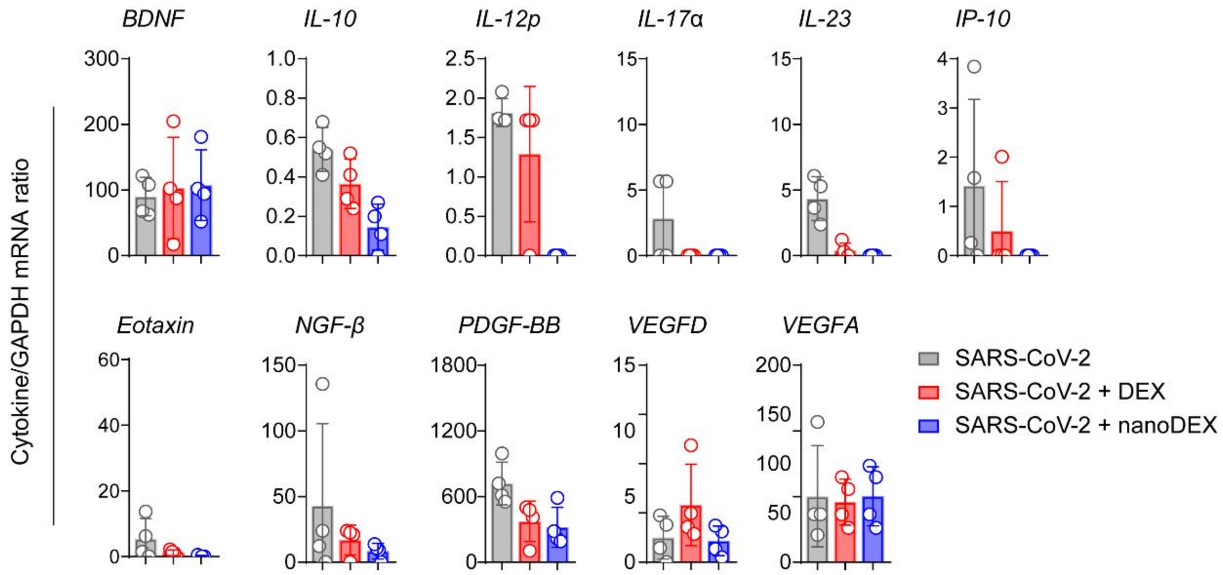

Supplementary Figure 27. qRT-PCR analysis of cytokine/chemokine expression in the serum at day 7 after indicated treatments. All data are presented as mean ± S.D. ($n = 4$).

Supplementary Table 3. Scoring of the inflammation observed in the H&E-stained trachea/lung sections in Figure 6C.

**Group**		SARS-CoV-2	SARS-CoV-2 + DEX	SARS-CoV-2 + nanoDEX
**Scoring**	**Trachea**	3	1	1
	**Lung**	3	1	1

The severity of inflammation observed in the H&E-stained lung/trachea sections was scored on a 0–3 scale defined as: 0 = no inflammatory response; 1 = mild inflammation with foci of inflammatory cells in bronchial or vascular wall and in alveolar septa; 2 = moderate inflammation with patchy inflammation or localized inflammation in walls of bronchi or blood vessels and alveolar septa, and less than one-third of the lung cross-sectional area is involved; 3 = severe inflammation with diffuse inflammatory cells in walls of bronchi or blood vessels and alveoli septa, and between one-third and two-thirds of the lung area is involved.

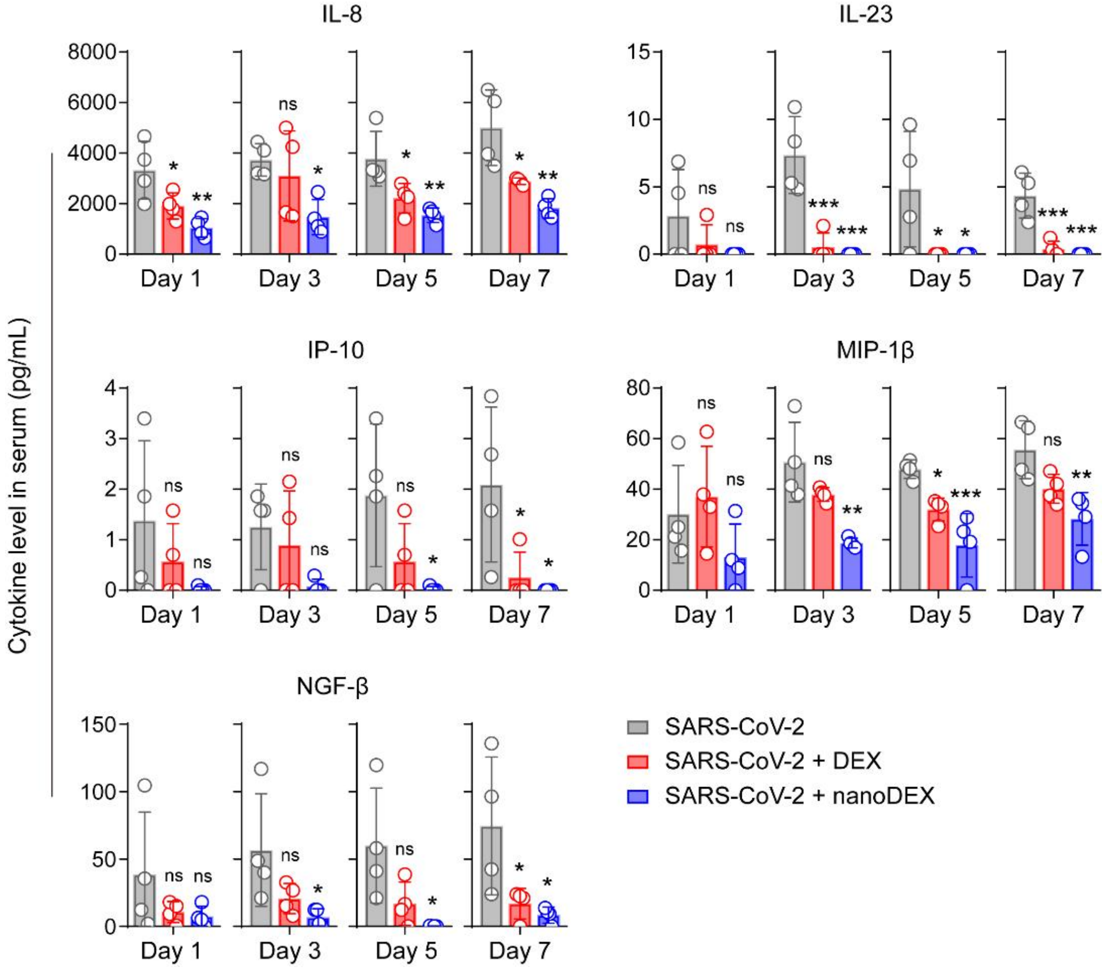

Supplementary Figure 28. Multiplex immunoassay analysis of cytokine/chemokine expression in the serum after indicated treatments. All data are presented as mean ± S.D. (n = 4).

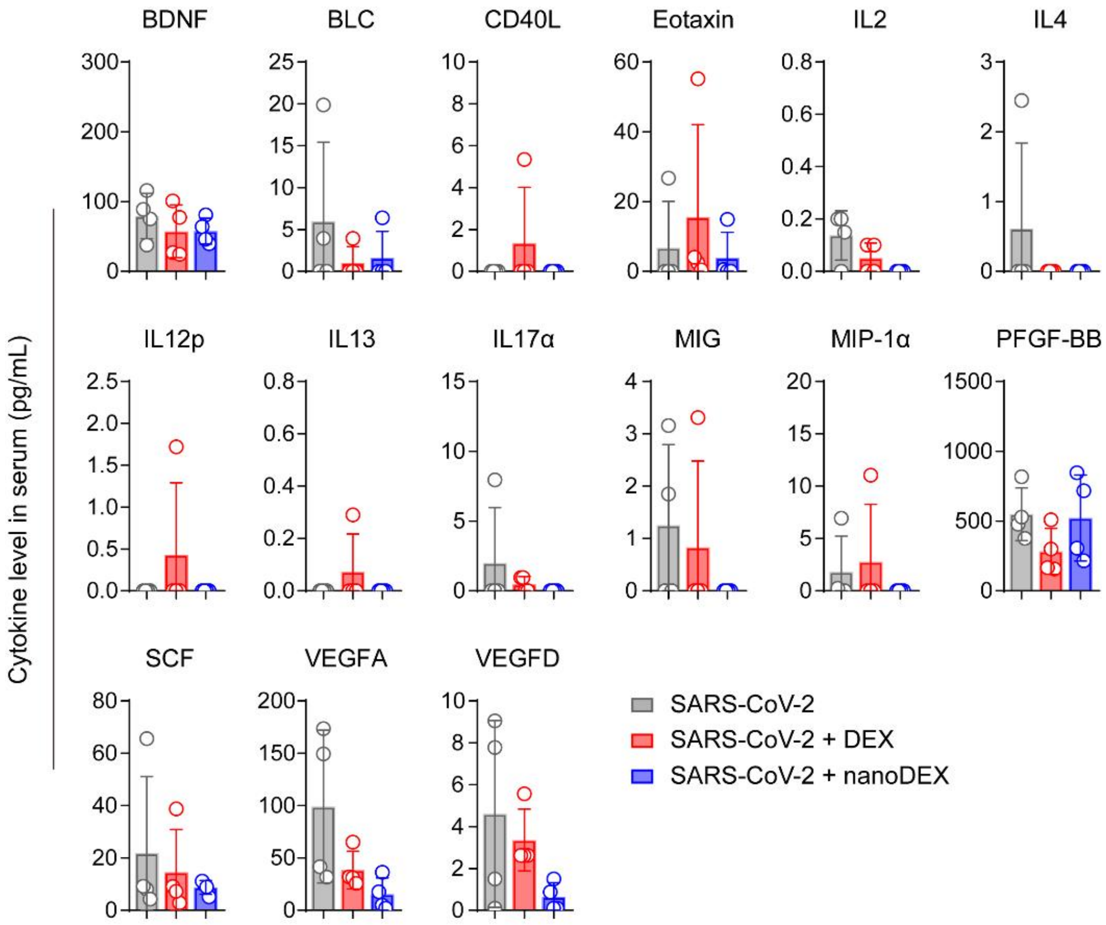

Supplementary Figure 29. Multiplex immunoassay analysis of cytokine/chemokine expression in the serum at day 1 after indicated treatments. All data are presented as mean ± S.D. (n = 4).

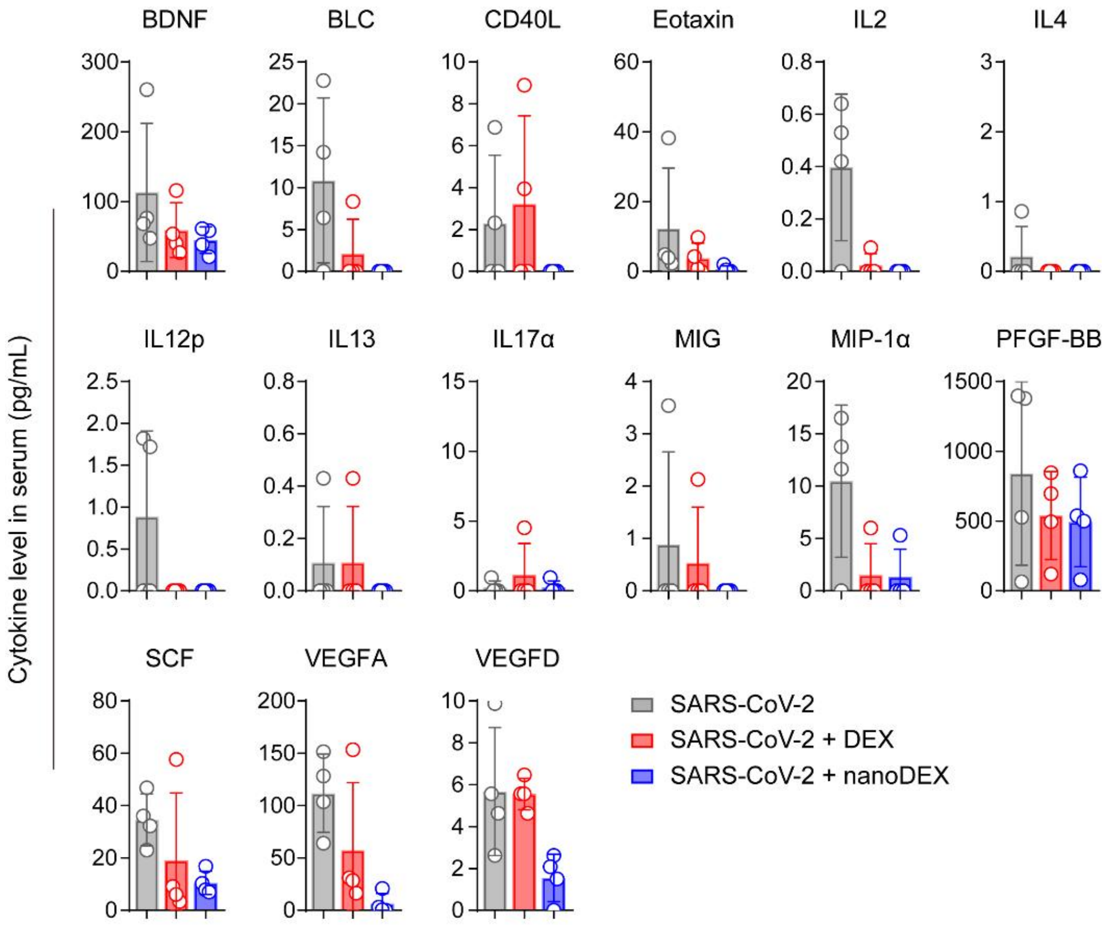

Supplementary Figure 30. Multiplex immunoassay analysis of cytokine/chemokine expression in the serum at day 3 after indicated treatments. All data are presented as mean ± S.D. ($n = 4$).

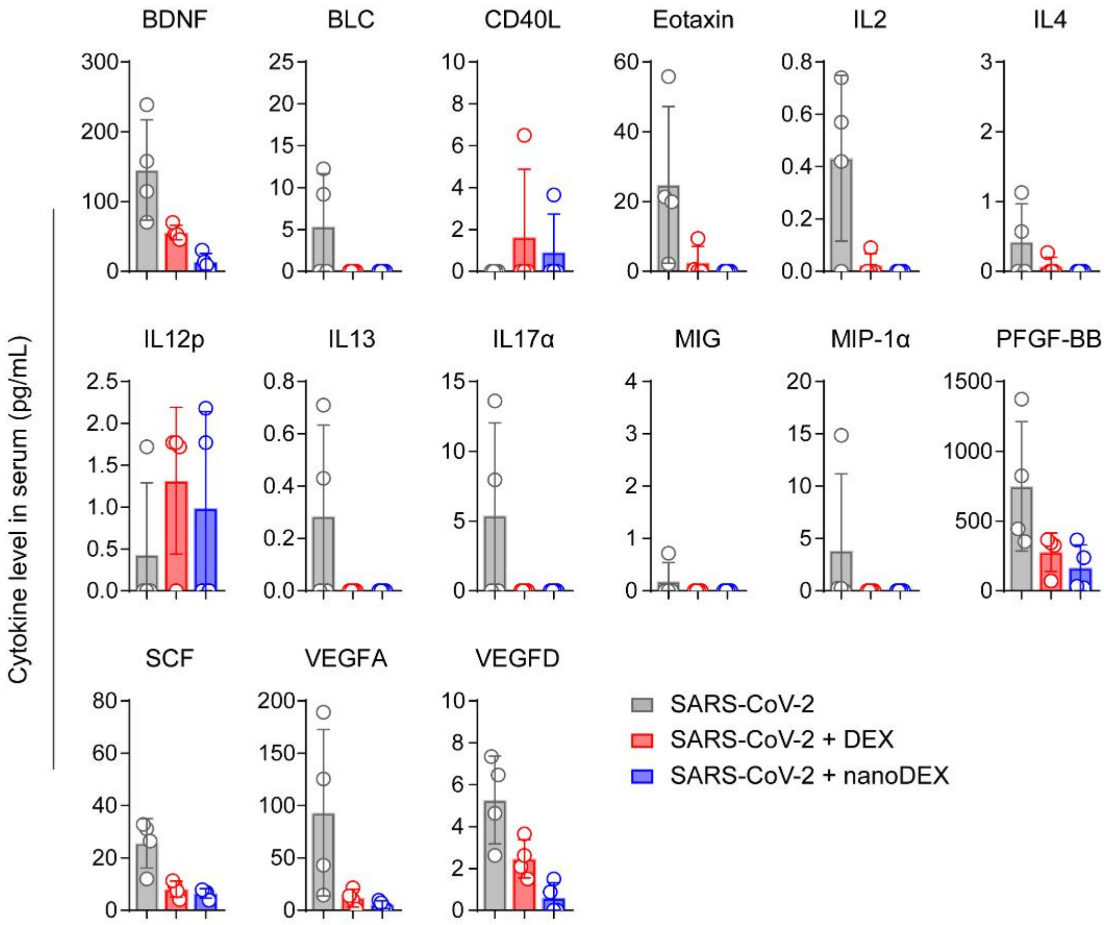

Supplementary Figure 31. Multiplex immunoassay analysis of cytokine/chemokine expression in the serum at day 5 after indicated treatments. All data are presented as mean ± S.D. (n = 4).

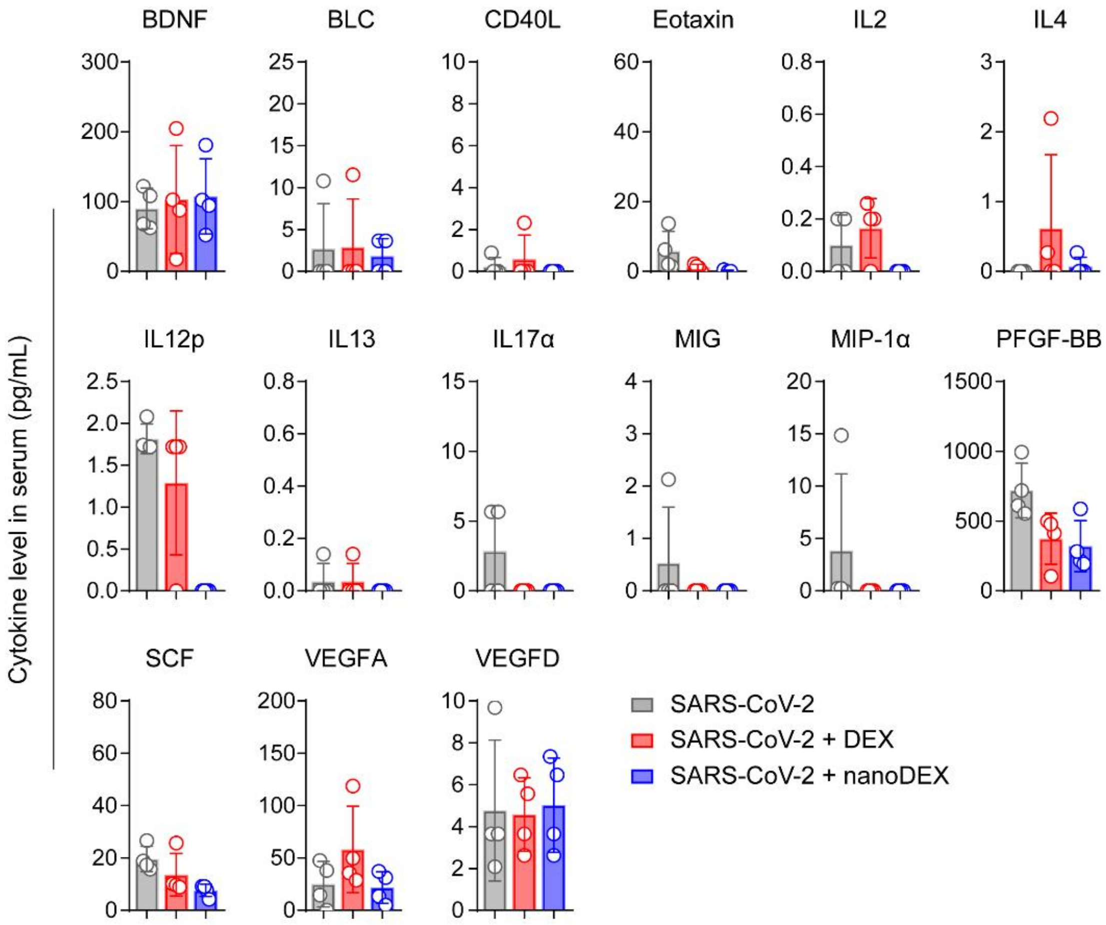

Supplementary Figure 32. Multiplex immunoassay analysis of cytokine/chemokine expression in the serum at day 7 after indicated treatments. All data are presented as mean ± S.D. ($n = 4$).

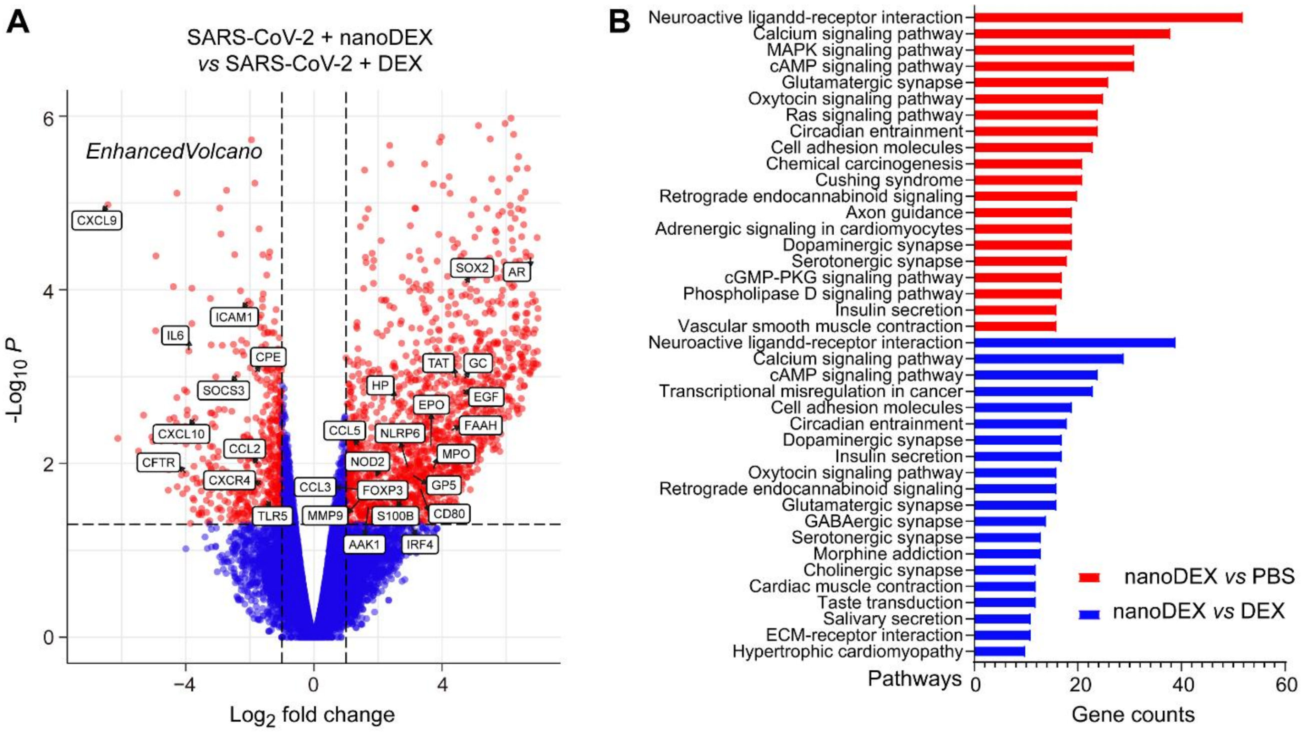

Supplementary Figure 33. Transcriptome analysis of the lung tissues of rhesus macaques after indicated treatments. (A) Volcano of differentially expressed genes by comparing the nanoDEX group to the DEX group. The genes associated with inflammation were marked by black boxes. (B) KEGG pathways in different groups.

Supplementary Table 4. The primers for qRT-PCR analysis of samples from mice.

mIL-1 alpha-F	CGAAGACTACAGTTCTGCCATT
mIL-1 alpha-R	GACGTTTCAGAGGTTCTCAGAG
mIL-1 beta-F	GCAACTGTTCCTGAACTCAACT
mIL-1 beta-R	ATCTTTTGGGGTCCGTCAACT
mIL-2-F	TGAGCAGGATGGAGAATTACAGG
mIL-2-R	GTCCAAGTTCATCTTCTAGGCAC
mIL-4-F	GGTCTCAACCCCCAGCTAGT
mIL-4-R	GCCGATGATCTCTCTCAAGTGAT
mIL-6-F	TAGTCCTTCCTACCCCAATTTCC
mIL-6-R	TTGGTCCTTAGCCACTCCTTC
mIL-10-F	GCTCTTACTGACTGGCATGAG
mIL-10-R	CGCAGCTCTAGGAGCATGTG
mTNF-alpha-F	CCCTCACACTCAGATCATCTTCT
mTNF-alpha-R	GCTACGACGTGGGCTACAG
mIL-12a-F	CAATCACGCTACCTCCTCTTTT
mIL-12a-R	CAGCAGTGCAGGAATAATGTTTC
mG-CSF-F	ATGGCTCAACTTTCTGCCCAG
mG-CSF-R	CTGACAGTGACCAGGGGAAC
mGM-CSF-F	GGCCTTGGAAGCATGTAGAGG
mGM-CSF-R	GGAGAACTCGTTAGAGACGACTT
mRANTES(Ccl5)-F	GCTGCTTTGCCTACCTCTCC
mRANTES(Ccl5)-R	TCGAGTGACAAACACGACTGC
mMCP-1(Ccl2)-F	TTAAAAACCTGGATCGGAACCAA
mMCP-1(Ccl2)-R	GCATTAGCTTCAGATTTACGGGT
mMIP-1 alpha(Ccl3)-F	TTCTCTGTACCATGACACTCTGC
mMIP-1 alpha(Ccl3)-R	CGTGGAATCTTCCGGCTGTAG
mSDF-1(Cxcl12)-F	TGCATCAGTGACGGTAAACCA
mSDF-1(Cxcl12)-R	TTCTTCAGCCGTGCAACAATC

mIP-10(Cxcl10)-F	CCAAGTGCTGCCGTCATTTTC
mIP-10(Cxcl10)-R	GGCTCGCAGGGATGATTTCAA
mEotaxin(Cxcl11)-F	GAATCACCAACAACAGATGCAC
mEotaxin(Cxcl11)-R	ATCCTGGACCCACTTCTTCTT
mMDC(Ccl22)-F	AGGTCCCTATGGTGCCAATGT
mMDC(Ccl22)-R	CGGCAGGATTTTGAGGTCCA
mKC(Ccl1)-F	CTGGGATTCACCTCAAGAACATC
mKC(Ccl1)-R	CAGGGTCAAGGCAAGCCTC
m6Ckine(Ccl21a)-F	GTGATGGAGGGGGTCAGGA
m6Ckine(Ccl21a)-R	GGGATGGGACAGCCTAAACT
mMIG(Cccl9)-F	GGAGTTCGAGGAACCCTAGTG
mMIG(Cccl9)-R	GGGATTTGTAGTGGATCGTGC
mGAPDH-F	TCAACAGCAACTCCCACTCTTCCA
mGAPDH-R	ACCCTGTTGCTGTAGCCGTATTCA

Supplementary Table 5. The primers for qRT-PCR analysis of samples from macaques.

monkey-IL1α-F	TTTGAAGACCTGAAGAACTGTTAC
monkey-IL1α-R	CAACCGTCTCTTCTTCAGAACCT
monkey-IL1B-F	TTGGAGCAACAAGTGGTGTTCT
monkey-IL1B-R	GAGAGGTGCTGATGTACCAGTT
monkey-IL2-F	GATTTACAGATGATTTTGAATGG
monkey-IL2-R	TGTGGCAATCCAATACAGGG
monkey-IL4-F	TGAACAGCCTCACAGAGCAG
monkey-IL4-R	AGCGAGTGTCCTTCTCATGG
monkey-IL6-F	CCAGCCACTGACCTCTTCAG
monkey-IL6-R	ACCAGGCAAGTGTCCTCATT
monkey-IL10-F	GATGCCTTCAGCAGAGTGAAG
monkey-IL10-R	GTCTGGGTCGTGGTTCTCAG
monkey-IL7-F	AGGTATATCTTTGGACTTCCTCC
monkey-IL7-R	AATTTCTTTCATGCTGTCCAATAAT
monkey-IL12β-F	ACTGATGTTTTAAAGGACCAG
monkey-IL12β-R	CTCTGCAGAGAGTGTAACGG
monkey-IL17-F	ACTCCTGGGAAGACCTCATTG
monkey-IL17-R	CTCTCAGGGTCCTCATTGCG
monkey-IFN-γ-F	GAAGCAGAAAACCTTAAGAAATAT
monkey-IFN-γ-R	CTCTGGTCATCTTTGAAGTTTT
monkey-TNF-α-F	AGAAGGCCTGTACCTCATCT
monkey-TNF-α-R	AGGGCAATGATCCCAAAGTAG
monkey-CSF3-F	GCCGCGCTGCAGGAGAAG
monkey-CSF3-R	GGAAGAGGCTGCTATGGAGT
monkey-GM-CSF-F	CCTGAGTAGAGACACTGCTG
monkey-GM-CSF-R	GTGCTGCTTGTAGTGGCTGG
monkey-RANTES-F	CTCTGCGCTCCTGCATCTG
monkey-RANTES-R	TCTCTGGGTTGGCACACA

monkey-MCP1-F	CAGGGGCTCGCTCAGCC
monkey-MCP1-R	CACTTCTGCTTGGGGTCAGC
monkey-MIP-1α-F	CTCTGCAACCGGATCTCAGC
monkey-MIP-1β-R	GACCTGCCGGCCTCTCTTG
monkey-MIP-1β-F	GCTCTCCAGCACTCTCAGC
monkey-MIP1B-R	CTCACTGGGGTCAGCGCAG
monkey-SDF1-F	CCGTCAGCCTGAGCTACAG
monkey-SDF1-R	GCTTTCTCCAGGTACTCCTG
monkey-IP10-F	TTTCTGACTCTAAGTGGTATTC
monkey-IP10-R	TTCTGGATTCAGACACCTCTT
monkey-MIG-F	TCTTCCTGGTTCTGATTGGAG
monkey-MIG-R	CATGTTTGATCTCCATTCTTCA
monkey-EOTAXIN-F	CTCACTGGGCCAGATTCTGTTG
monkey-EOTAXIN-R	ATGGAATCCTGCACCCACTTC
monkey-TARC-F	GCTTCTCTGCAGCACATCCATG
monkey-TARC-R	CCGAACAGATGGCCTTGTTC
monkey-KC-F	CAGGCAGCCTCCATGCAGC
monkey-KC-R	CTTAGATCTGGGGCTGTCC
monkey-6Ckine-F	CTTTGGCATCCCCGGGAC
monkey-6Ckine-R	GGTCTGCACATAGCTCTGCC
monkey-MDC-F	TGGCGCTTCAAGCAACTGAG
monkey-MDC-R	CACTCTGGGATCGGCACAGA
monkey-GAPDHF	AGCCCCATCACCATCTTCC
monkey-GAPDHR	AATGAGCCCCAGCCTTCTC
